# Supplementary material for: Haprolid Inhibits Tumor Growth of Hepatocellular Carcinoma through Rb/E2F and Akt/mTOR Inhibition
Source: Cancers (Basel). 2020 Mar 6;12(3):615. doi: 10.3390/cancers12030615 (PMC7139901; doi:10.3390/cancers12030615)
Supplement: Supplementary file 1 [file cancers-12-00615-s001.zip › cancers-670532 supplementary final/cancers-670532 supplementary proof.pdf]

Supplementary Materials and Methods

Determination of Cell Viability and Proliferation using WST-1 Assay

A total $2x10^3$ cells with 200 µl complete media were seeded per well into 96-well plates. PBS was pipetted into the surrounding wells to keep the media from drying. The media were refreshed on the second day and the HCC cells were treated with DMSO or Haprolid (0.006, 0.06, 0.6 and 6 µg/ml). Following the indicated treatment HCC cells were further incubated for additional time points of 24 hrs, 48 hrs, 72 hrs and 96 hrs respectively. Further, 20 µl of WST-1 reagent (Roche Diagnostics, Mannheim, Germany) was added per well and incubated for another 2 hours at 37°C before reading. The plates were well mixed and read at a wavelength of 450 nm with a reference wavelength of 690 nm according to the manufacturer's datasheet using a Microplate Reader Synergy™ HT (BioTek, Bad Friedrichshall, Germany).

Crystal Violet Staining

HCC cells were plated at a density of $2x10^5$ cells per well in a 6-well plate. For each condition the cells were treated in triplicate wells. For observation, the media were aspirated and the cells were washed twice with cold PBS. The HCC cells were fixed with ice cold 100% methanol (AppliChem, Darmstadt, Germany) for 10 mins and stained with 0.5% crystal violet for 10 mins. Excess crystal violet solution was removed and the cells were washed with cold PBS. The plates were dried at room temperature. Images were taken using a digital camera.

Migration Assay

HCC cells were seeded in a 6-well plate and left to reach 80% confluence. Initially, cells were starved for 24 hrs in media containing 2% FBS in order to minimize the

interference of cell proliferation. Then the cells were further incubated for 48 hrs in the starvation media containing either the controls (DMSO) or Haprolid. Afterwards a scratch was done using a 200 µl pipette tip under an angle of around 30 degrees for each treatment. Then cells were washed with pre-warmed PBS and snapshot pictures were taken using a regular inverted microscope (Carl Zeiss, Göttingen Germany). Cells were incubated for an additional 24 hrs after which photographs were taken for the wounded area. The migration index was calculated using the following formula:

$$Migration\ Index = \frac{Width\ of\ the\ wound_{0h} - Width\ of\ the\ wound_{24h}}{Width\ of\ the\ wound_{0h}} \times 100$$

Invasion Assay

The invasion assay was performed using a specialized invasion chamber BioCoat™ Matrigel Invasion Chamber from CORNING, Bedford, USA. Briefly a total of 2 $x10^4$ cells were plated in the upper chamber with 0.5 ml serum free media. Simultaneously the cells were treated with Haprolid (0.06, 0.6 and 6 µg/ml) or DMSO. These upper chambers were carefully placed into the 24-well plate containing media supplemented with 20% FBS as a chemoattractant. After 48 hrs incubation at 37°C, 5% CO_2 atmosphere the cells on the upper surface of the membrane were mechanically removed with cotton swab. The invading cells were fixed 2 mins in 100% ice-cold methanol (AppliChem, Darmstadt, Germany) and stained 2 mins with 1% toluidine blue (Sigma-Aldrich, Schnelldorf, Germany) in 1% borax (AppliChem, Darmstadt, Germany). After staining the membrane was removed from the insert and placed on a microscope slide. Cells were then counted under the microscope (OLYMPUS,

Hamburg, Germany) at 200 X magnifications. The following calculation of the invasion index was done according to the manufacturer’s protocol:

$$Invasion\ Index = \frac{\%\ Invasion\ Test\ Cell}{\%\ Invasion\ Control\ Cell}$$

Three-Dimensional (3D) Tumor Spheroid Growth and Invasion Assay

HCC cells were cultured in hanging drops using the lid of TC Dish 100 Standard (Sarstedt, Nümbrecht, Germany). 3000 cells were seeded in 20 ul drop and cultured for 7 days to obtain spheroids around 200 μm in diameter. Collect the spheroids and allow 10 mins for the spheroids to settle down at the bottom of the Eppendorf microcentrifuge tube. Mix 100 μl of the basement membrane materials Matrigel Matrix (CORNING, Bedford, USA) with 100 μl of cold (4 °C) type I collagen (Sigma-Aldrich, Schnelldorf, Germany) in a separate pre-chilled tube. Aspirate the spheroids from the 40 μl bottom portion of the microcentrifuge tube and combine with the basement membrane materials/type I collagen mixture. Place the plate into a 37 °C incubator and leave undisturbed for 30 mins to polymerize the 3D cultures. Slowly submerge the 3D cultures in 1 ml of warm cell culture media. Image the spheroids at indicated time-points using an inverted microscope. Quantitate spheroid growth and invasion ability using image analysis software Image J 1.47 (Wayne Rasband, National Institutes of Health, USA).

Western Blot Analysis

Treated cells were harvested and proteins were extracted using lysis buffer [50 mM Tris, 80 mM NaCl, 1 mM EDTA, 1 mM EGTA and Protease Inhibitor Cocktail Tablets (Roche, Mannheim, Germany)]. Protein concentrations were determined by DC protein assay kit (BIO-RAD, München, Germany) following the manufacturer’s instruction. Lysates containing 10 μg protein were mixed with loading buffer and

boiled for 5 mins at 95°C before being separated on SDS-polyacrylamide gels. The gels were run at 65 and 120 V (BIO-RAD, München, Germany). After the transfer to the PDVF membranes (Merck, Darmstadt, Germany) at 90 V for 1 hr, the blots were blocked with 5% non-fat milk (AppliChem, Darmstadt, Germany) or with 5% bovine serum albumin (BSA) (Carl Roth, Karlsruhe, Germany) for 1 to 3 hrs at room temperature. These membranes were further probed with primary antibodies overnight N-cadherin (1:1000; Cell signaling, #13116), E-cadherin (1:1000; Cell signaling, #3195), Vimentin (1:1000; Santa Cruz Biotechnology, sc-7557), Snail (1:1000; Cell signaling, #3879), β-Actin (1:1000; Santa Cruz Biotechnology, sc-47778), Cyclin A (1:1000; Santa Cruz Biotechnology, sc-751), Cyclin B1(1:1000; Santa Cruz Biotechnology, sc-245), PAPR (1:1000; Cell signaling, #9542), p-Rb(1:1000; Cell signaling, #9308), Rb (1:1000; Cell signaling, #9313), E2F-1 (1:1000; Cell signaling, #3742), CDK2 (1:1000; Cell signaling, #2546),p-Histone H3 (1:1000; Cell signaling, #3377), p21 (1:500; Santa Cruz Biotechnology, sc-397), p27 Kip1 (1:1000; Cell signaling, #2552), p-Akt (1:1000; Cell signaling, #9271), Akt (1:1000; Cell signaling, #4685), p-mTOR (1:1000; Cell signaling, #2971), mTOR (1:1000; Cell signaling, #2983), p-p70 S6 Kinase(1:1000; Cell signaling, #9205), p-S6 Ribosomal Protein (1:1000; Cell signaling, #5364), S6 Ribosomal Protein(1:1000; Cell signaling, #2217) and p-Erk1/2 (1:1000; Cell signaling, #4370). The signal was detected by Amersham Hyperfilm^{TM} ECL (GE Healthcare Limited, Buckinghamshire, UK).

Cell Cycle Assay

To figure out the cell cycle distribution after Haprolid treatment, $2x10^5$ cells were seeded in a 6-well plate and treated as indicated. At certain time points, cells were harvested in the appropriate manner and washed with PBS and then fixed with 70% ice cold ethanol in -20°C. Alcohol-fixed cells are stable for several weeks at -20°C.

These cells were further centrifuged and washed with cold PBS. Then cells were stained with 10µg/ml of Propidium iodide (Sigma Aldrich, Schnelldorf, Germany) and 50µg/ml Ribonuclease (Sigma Aldrich, Schnelldorf, Germany) in PBS and incubated for at least 45mins at room temperature in dark. The signal was detected using Flow Cytometer FACS Canto II (BD Biosciences, Heidelberg, Germany) and was analyzed using FlowJo Version 10 software (FlowJo, LLC, Ashland, USA).

Detection of Apoptosis by Annexin V Staining

To determine the apoptosis, cells were seeded ($2x10^5$) in a 6-well plate and were incubated overnight at 37°C. These cells were further treated with Haprolid (0.06, 0.6 and 6 µg/ml) or DMSO for 48 and 96 hrs. After the respective treatments, the supernatant (including floating apoptotic cells) was collected and the adherent cells were trypsinized and then collected to the same tube. The tubes were centrifuged for 5 mins at 1000 rpm. The supernatant was discarded, the cell pellet was suspended with 1 ml 1 X Binding Buffer at a concentration of approximately 1×10^6 cells/ml on ice and were stained with FITC Annexin V and PI according to the manufacture's instruction using FITC Annexin V Apoptosis Detection Kit I (BD Biosciences, Heidelberg, Germany). The signal was detected using Flow Cytometer FACS Canto II (BD Biosciences, Heidelberg, Germany) and analyzed using FlowJo Version 10 software (FlowJo, LLC, Ashland, USA).

Immunohistochemistry Staining

Formalin-fixed, paraffin sections were deparaffinized in histol (Roth, Karlsruhe, Germany) and rehydrated in ethanol (100%, 95%, 75% and 40% ethanol). The sections were then subjected to antigen retrieval by heating the slides in a pressure cooker with Antigen Unmasking Solution (Vector Laboratories, Inc., Burlinghame,

USA) then rinsed in PBS. The specimens were then incubated for 10 mins in 1% H_2O_2 to inactivate endogenous peroxidase activity and further were blocked 1 hr in blocking solution (5% normal serum in 0.3% Triton X-100) at room temperature to avoid non-specific binding. Hybridization with the primary antibody (1:100) was carried out overnight at 4°C. The next day, the primary antibody was rinsed by PBS and the slides were incubated with secondary antibody (1:200) for 1 hr. The manufacturer's protocols were used for ABC and DAB substrates (Vector Laboratories, Inc., Burlinghame, USA). Then, the slides were counterstained with hematoxylin for 1 min and dehydrated in 40%, 75%, 95% and 100% ethanol. Finally, slides were cleared with histol and mounted with mounting medium (Vector Laboratories, Inc., Burlinghame, USA). The histology was reviewed by professional pathologist. Images were taken by light microscope (OLYMPUS, Hamburg, Germany) and positive cells were quantified using software Image J.

Table. S1. The percentages of the cell cycle distribution across the sub-G1-, G1-, S- and G2-M-phases in control- and Haprolid-treated HCC cells

Hep3B (%)				
48h	sub-G1	G1	S	G2-M
DMSO	5,60	60,15	12,64	21,61
0.06µg/ml	7,65	64,94	7,61	19,80
0.6µg/ml	9,29	65,83	7,99	16,89
6µg/ml	14,93	62,19	6,71	16,16
96h	sub-G1	G1	S	G2-M
DMSO	5,02	56,20	14,00	24,78
0.06µg/ml	12,17	59,00	7,89	20,94
0.6µg/ml	31,03	50,44	5,79	12,74
6µg/ml	31,20	47,05	7,22	14,53
Huh-7 (%)				
48h	sub-G1	G1	S	G2-M
DMSO	2.33	57.48	21.30	18.89
0.06µg/ml	6.63	56.95	16.30	20.12
0.6µg/ml	7.16	60.34	14.28	18.22
6µg/ml	4.67	64.60	14.96	15.77
96h	sub-G1	G1	S	G2-M
DMSO	3.08	54.40	19.57	22.95
0.06µg/ml	4.38	57.57	17.91	20.14
0.6µg/ml	14.53	55.28	12.30	17.89
6µg/ml	8.92	59.90	13.65	17.52
HepG2 (%)				
48h	sub-G1	G1	S	G2-M
DMSO	0.65	52.3	14	31.1
0.06µg/ml	1.31	59.5	6.69	31.2
0.6µg/ml	0.91	57.4	5.94	34.2
6µg/ml	1.32	59.8	5.8	31.6
96h	sub-G1	G1	S	G2-M
DMSO	1.09	54.8	12.1	30.3
0.06µg/ml	2.45	66.6	8.07	22.3
0.6µg/ml	2.11	58.1	5.73	32
6µg/ml	2.37	59.7	4.29	31.8

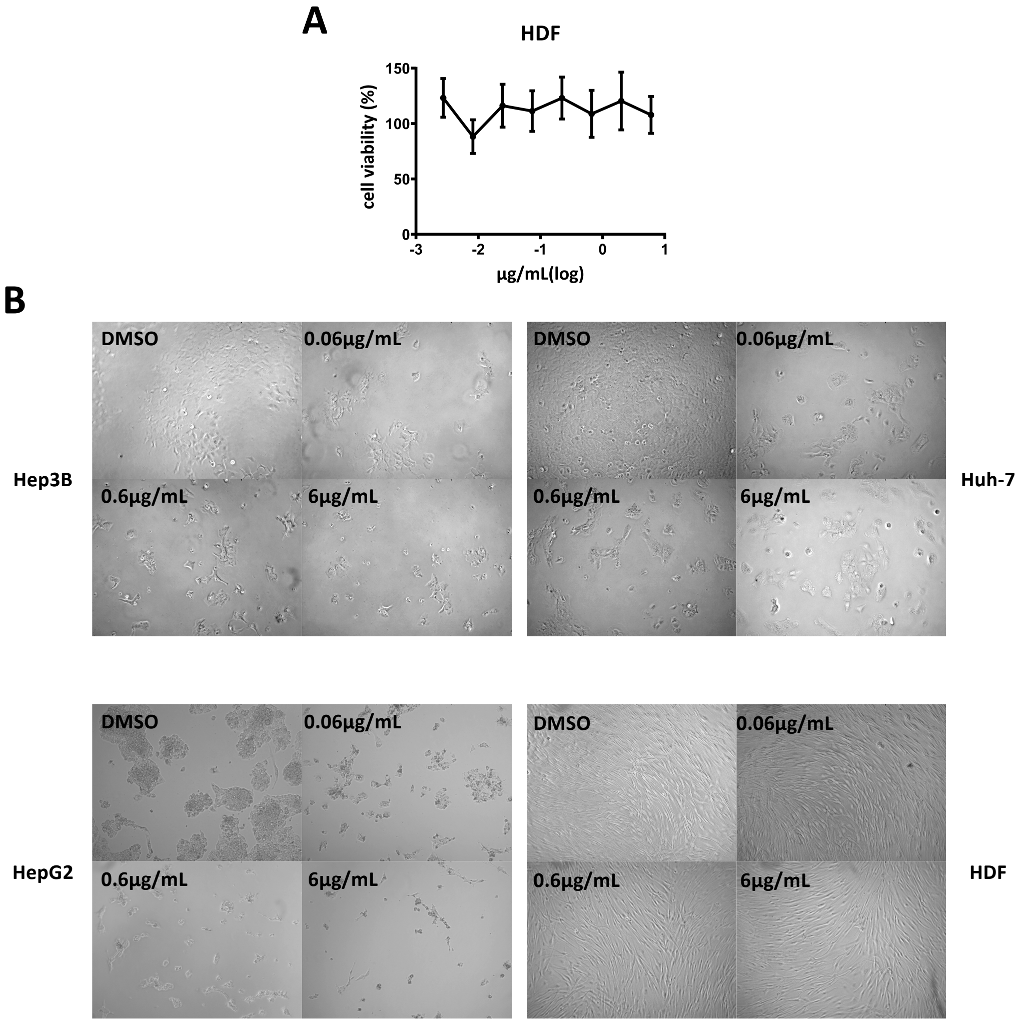

Figure. S1. Differential cytotoxicity between HCC cells and human fibroblast cells. (A) HDF (human dermal fibroblast) cells were treated with increasing concentration of Haprolid (0,001 to 18 µg/ml) for 96 hrs. WST-1 assay was performed to analyze cellular viability, DMSO was used as negative control. (B) Hep3B, Huh-7, HepG2 and HDF cells were seeded in 96-well plates and treated with Haprolid (0.06, 0.6 and 6 µg/ml) or DMSO and corresponding light microcope pictures (100 X magnification) show treatment results after 96 hrs. Data represent means ± SEM of at least three independent experiments.

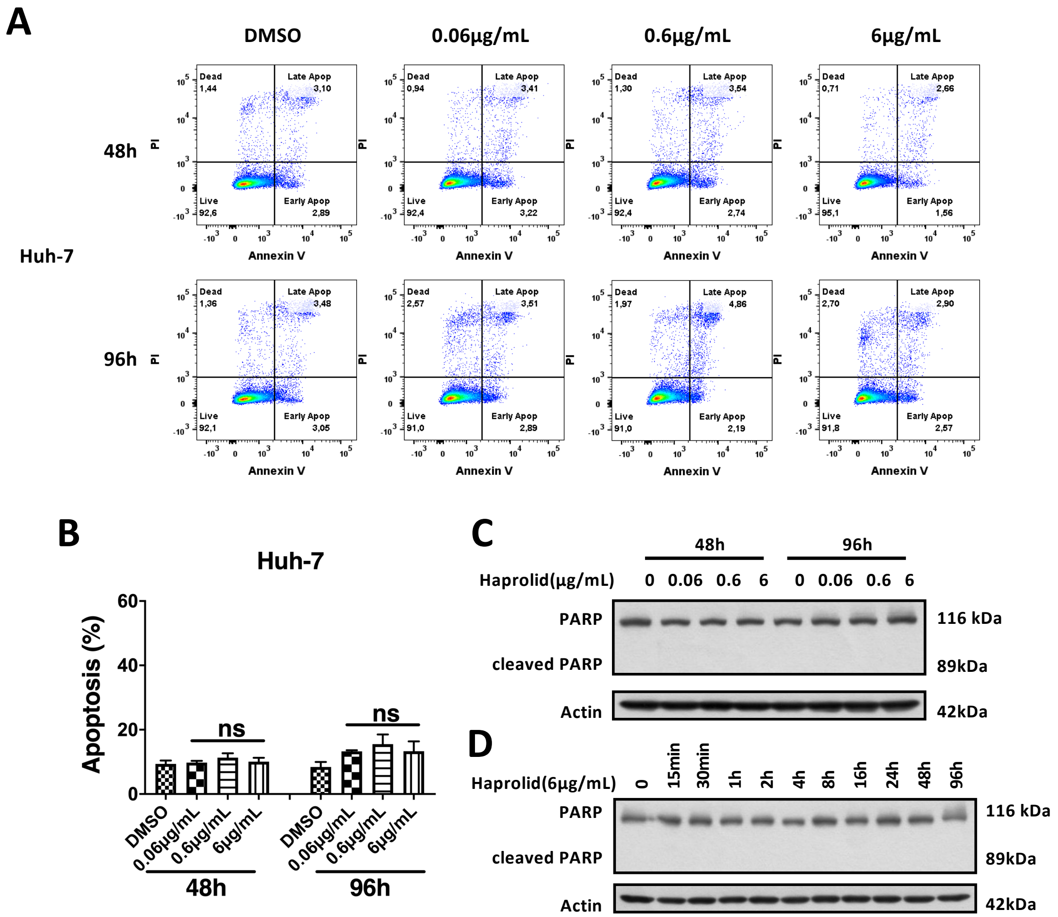

Figure. S2. Apoptotic effect of Haprolid on Huh-7 cells. (A) Huh-7 cells were treated with DMSO or Haprolid (0.06, 0.6 and 6 μg/ml) for 48 hrs and 96 hrs, the level of apoptosis was measured by staining with Annexin V and PI using flow cytometry. Representative FACS measurements are presented. (B) The apoptotic positive cells were calculated and plotted in bar graph. (C) Expression of PARP and cleaved PARP (a marker for apoptosis) was measured by western blotting. (D) Kinetic analysis of PARP cleavage was performed by western blotting. Data represent means ± SEM of at least three independent experiments.

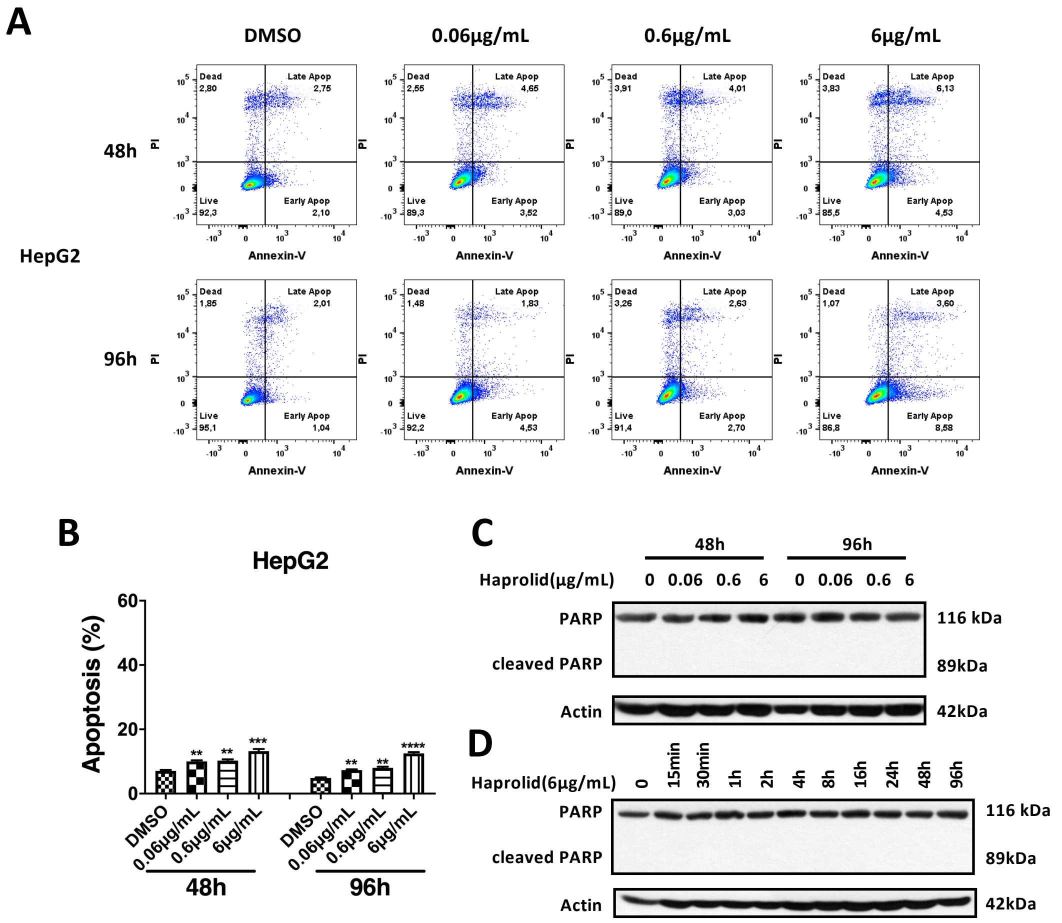

Figure. S3. Apoptotic effect of Haprolid on HepG2 cells. (A) HepG2 cells were treated with DMSO or Haprolid (0.06, 0.6 and 6 μg/ml) for 48 hrs and 96 hrs, the level of apoptosis was measured by staining with Annexin V and PI using flow cytometry. Representative FACS measurements are presented. (B) The apoptotic positive cells were calculated and plotted in bar graph. There is significant apoptosis induction compared with untreated group but the percentages of apoptotic cells are very limited. (C) Expression of PARP and cleaved PARP (a marker for apoptosis) was measured by western blotting. (D) Kinetic analysis of PARP cleavage was performed by western blotting. Data represent means ± SEM of at least three independent experiments. $^{**}P < 0.01$, $^{***}P < 0.001$, $^{****}P < 0.0001$.

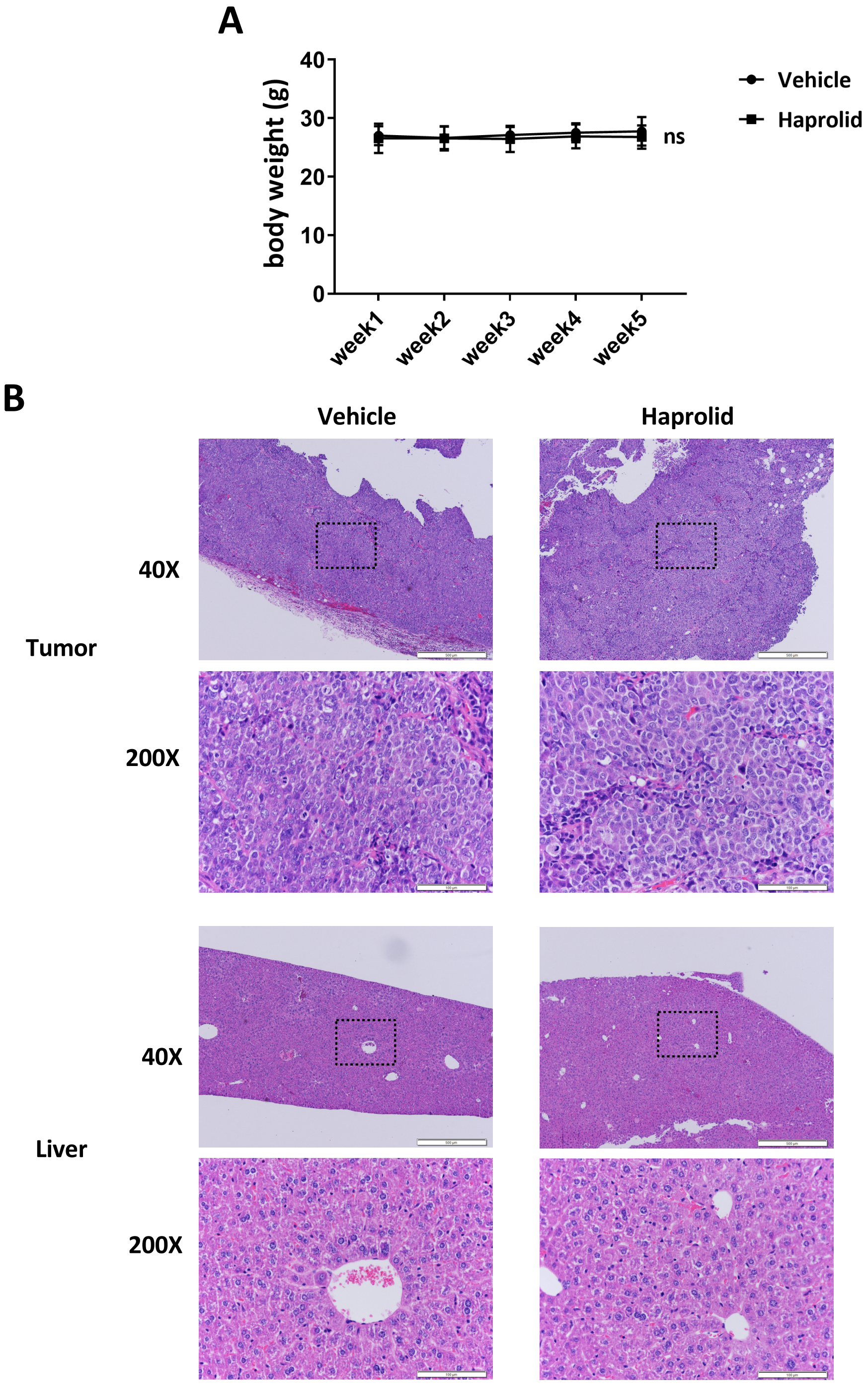

Figure. S4. Evaluation of side effects of Haprolid in mice. (A) Body weight of control- and Haprolid-treated mice. Both groups showed stable body weight throughout the experiment. (B) H&E stainings of tumors and livers from control and Haprolid-treated mouse groups. Scale bars: 100 µm for 200 X magnification; 500 µm for 40 X magnification.

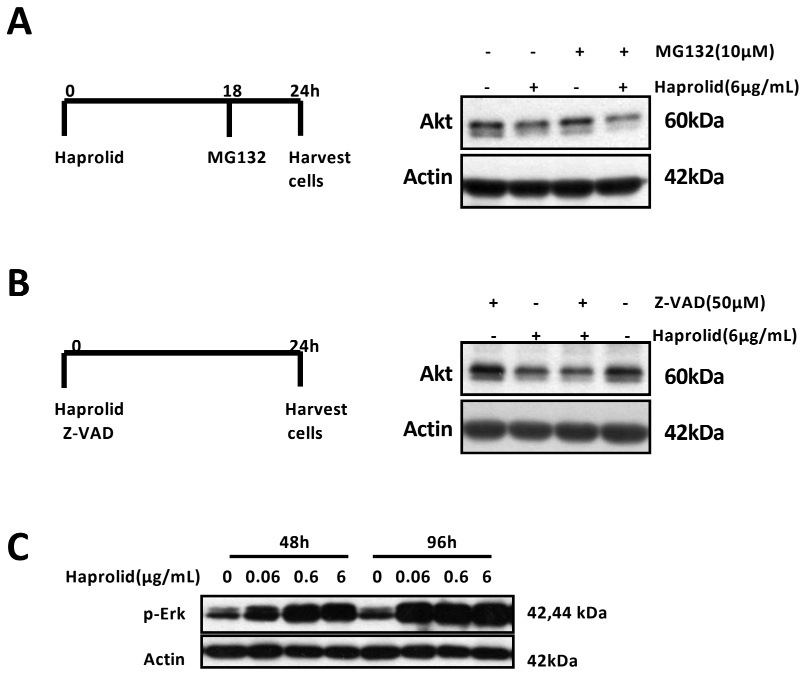

Figure. S5. Haprolid-induced degradation of Akt protein is neither proteasome nor caspase dependent and Erk phosphorylation is significantly increased by Haprolid treatment. (A) Hep3B cells were treated with 6 μg/ml Haprolid for 24 hrs and 10 μM of the proteasome inhibitor MG132 (MG) were added to the samples 6 hrs before harvesting the cells. Whole-cell extracts were used to assess total Akt levels by immunoblotting. (B) Hep3B cells treated with 6 μg/ml Haprolid alone or in combination with 10 μM of a general caspase inhibitor, Z-VAD, for 24 hrs. Whole-cell extracts were used to evaluate total Akt protein level by immunoblotting. (C) Hep3B cells were treated with increasing concentrations of Haprolid for 48 hrs and 96 hrs. Cell lysates were analyzed by immunoblotting for p-Erk expression.
