# Supplementary figures and images for: Haprolid Inhibits Tumor Growth of Hepatocellular Carcinoma through Rb/E2F and Akt/mTOR Inhibition
Source: Cancers (Basel). 2020 Mar 6;12(3):615. doi: 10.3390/cancers12030615 (PMC7139901; doi:10.3390/cancers12030615)

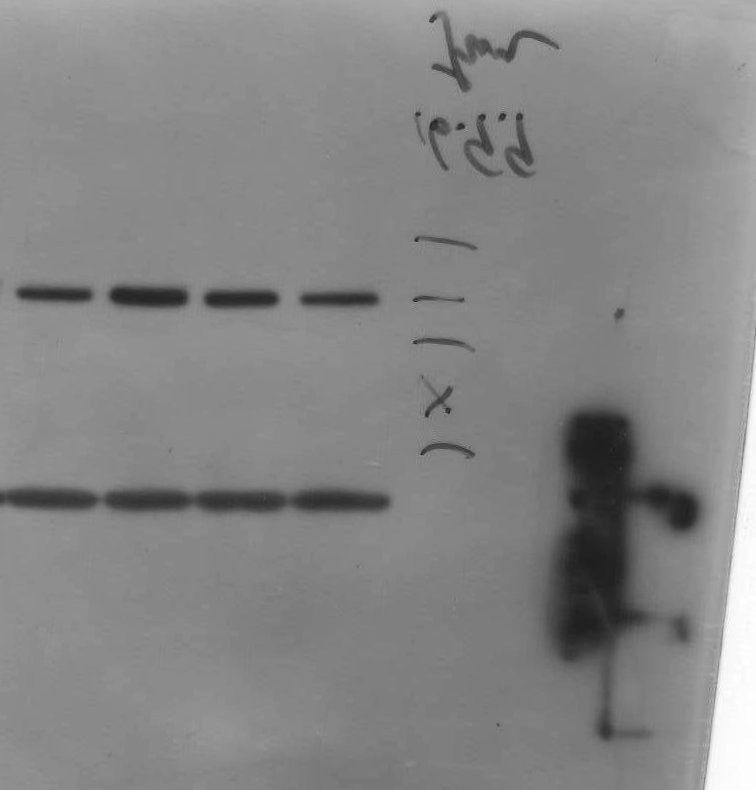

Supplement: Supplementary file 1 [file cancers-12-00615-s001.zip › cancers-670532 supplementary final/Western Blot/2016-09-09 huh -7 E-cads.jpg]

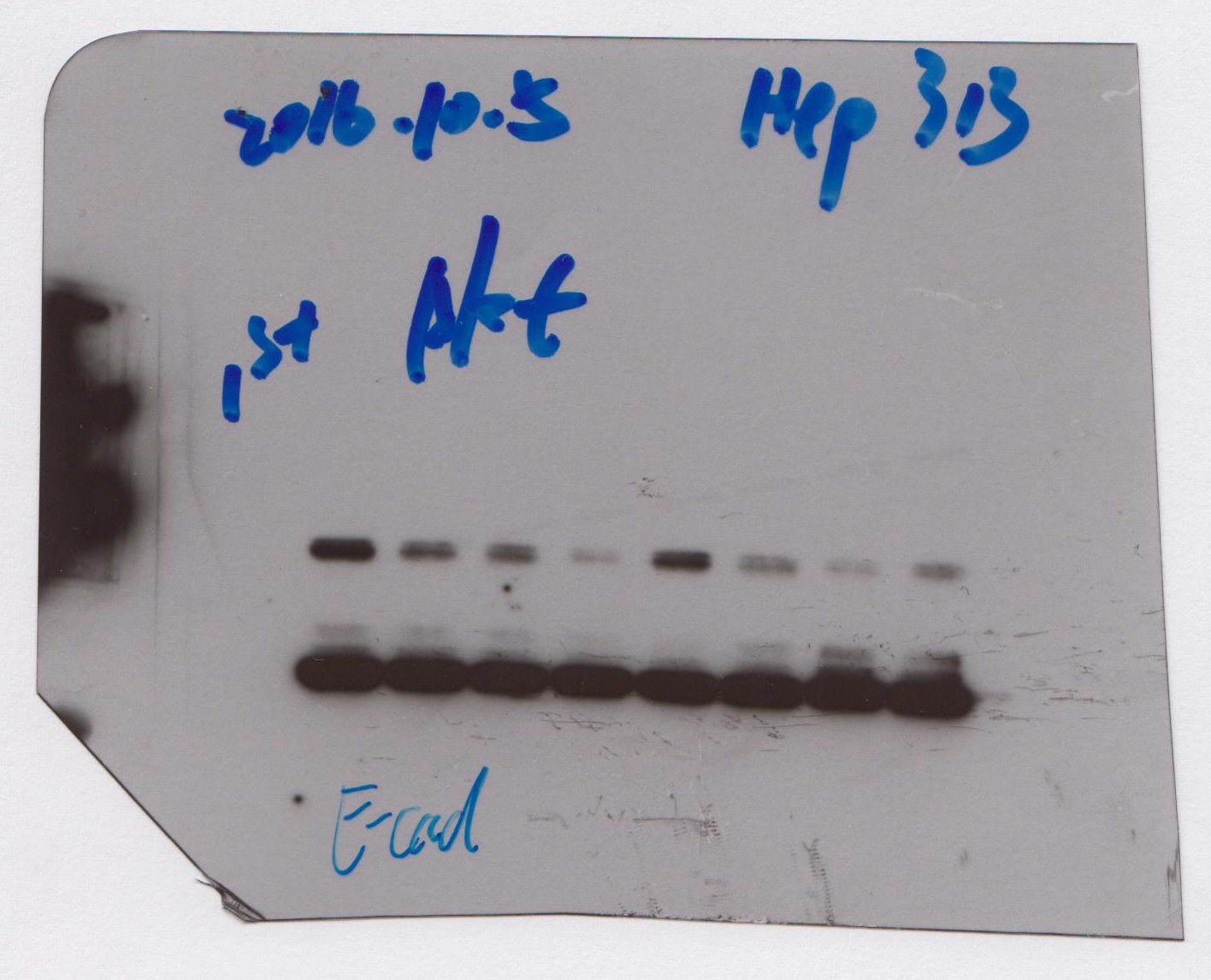

Supplement: Supplementary file 1 [file cancers-12-00615-s001.zip › cancers-670532 supplementary final/Western Blot/2016-10-05 akt HEP 3B.jpg]

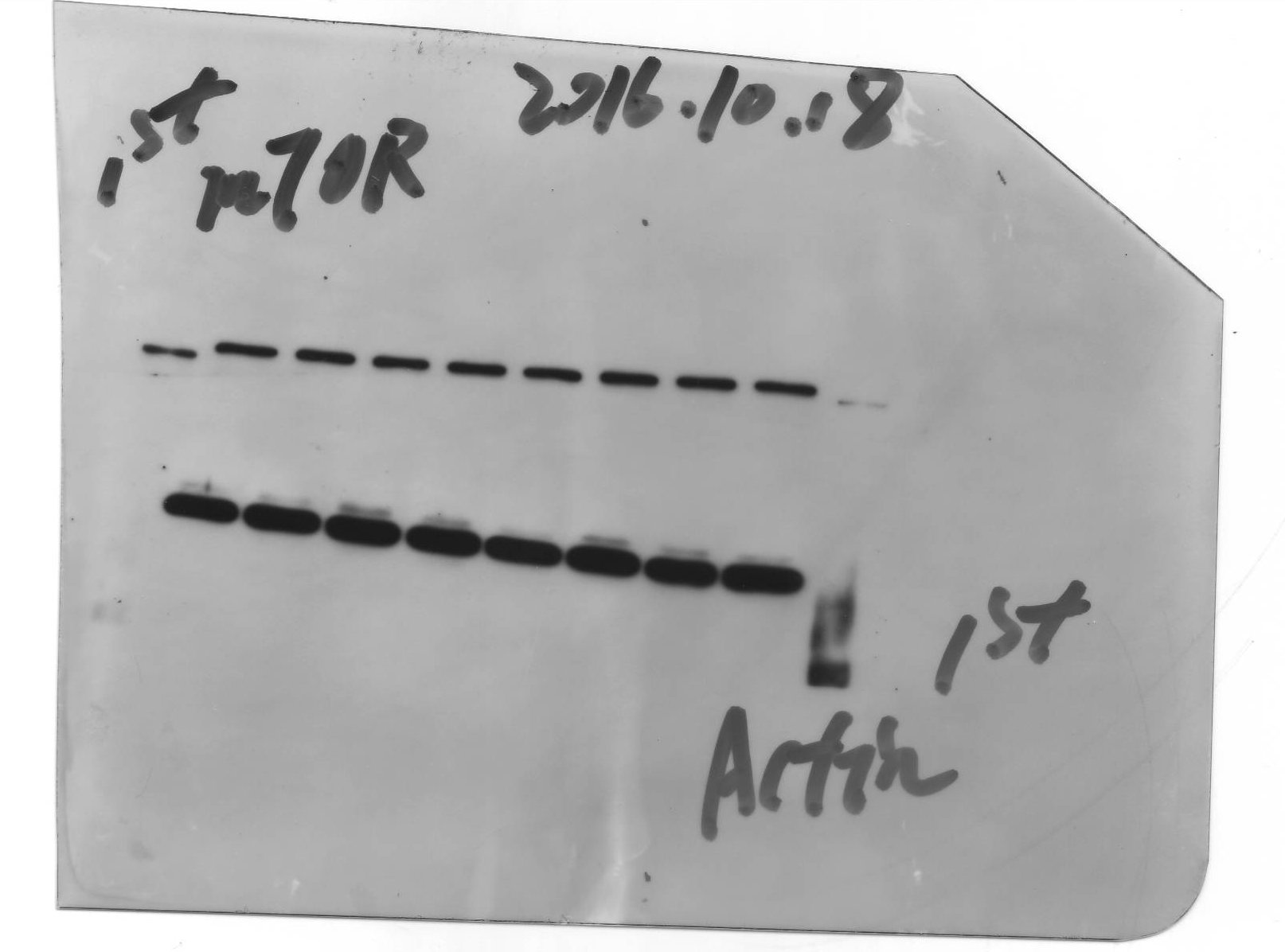

Supplement: Supplementary file 1 [file cancers-12-00615-s001.zip › cancers-670532 supplementary final/Western Blot/2016-10-18 Actin+mTOR.jpg]

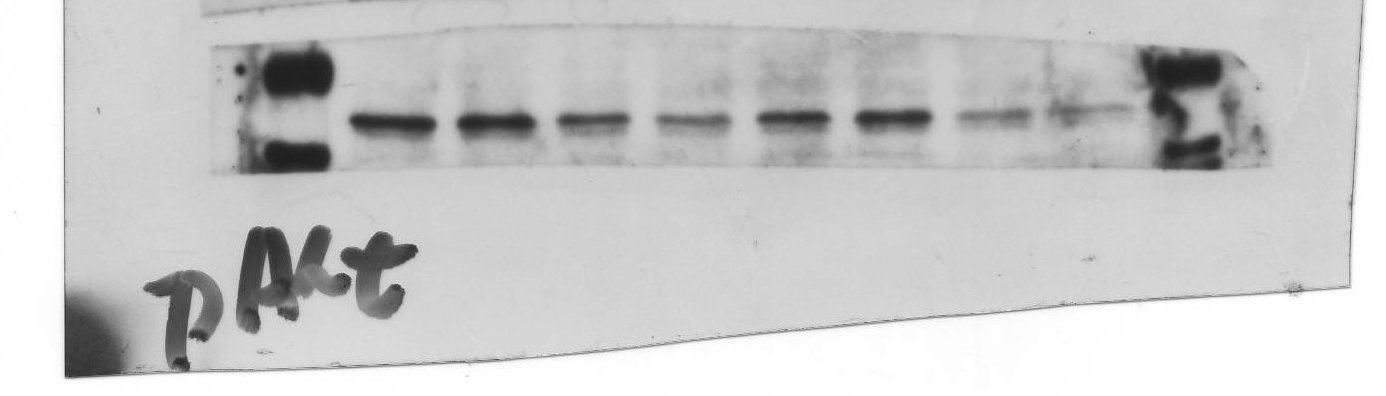

Supplement: Supplementary file 1 [file cancers-12-00615-s001.zip › cancers-670532 supplementary final/Western Blot/2016-10-18 hep 3b p-Akt.jpg]

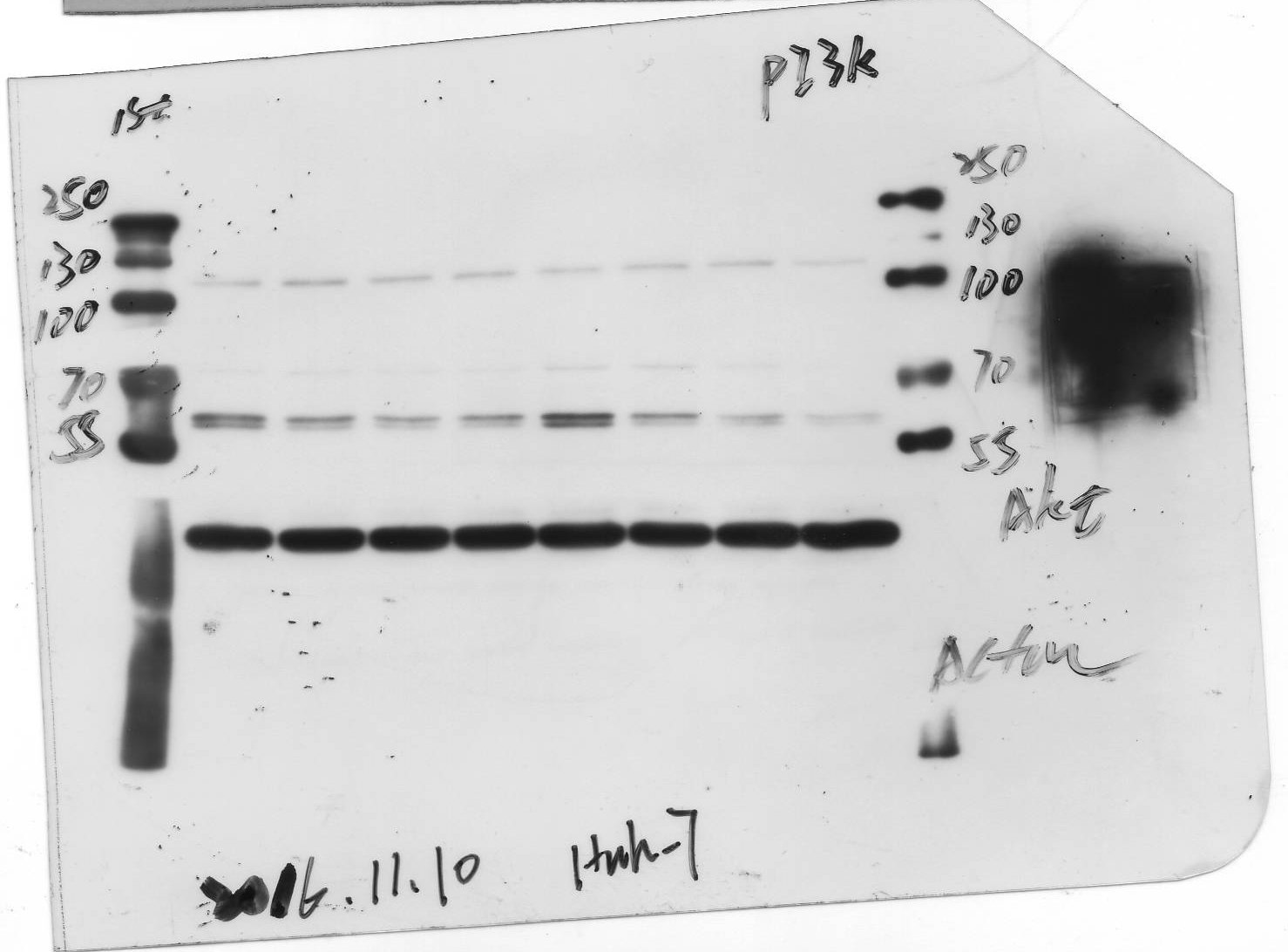

Supplement: Supplementary file 1 [file cancers-12-00615-s001.zip › cancers-670532 supplementary final/Western Blot/2016-11-10 akt huh-7.jpg]

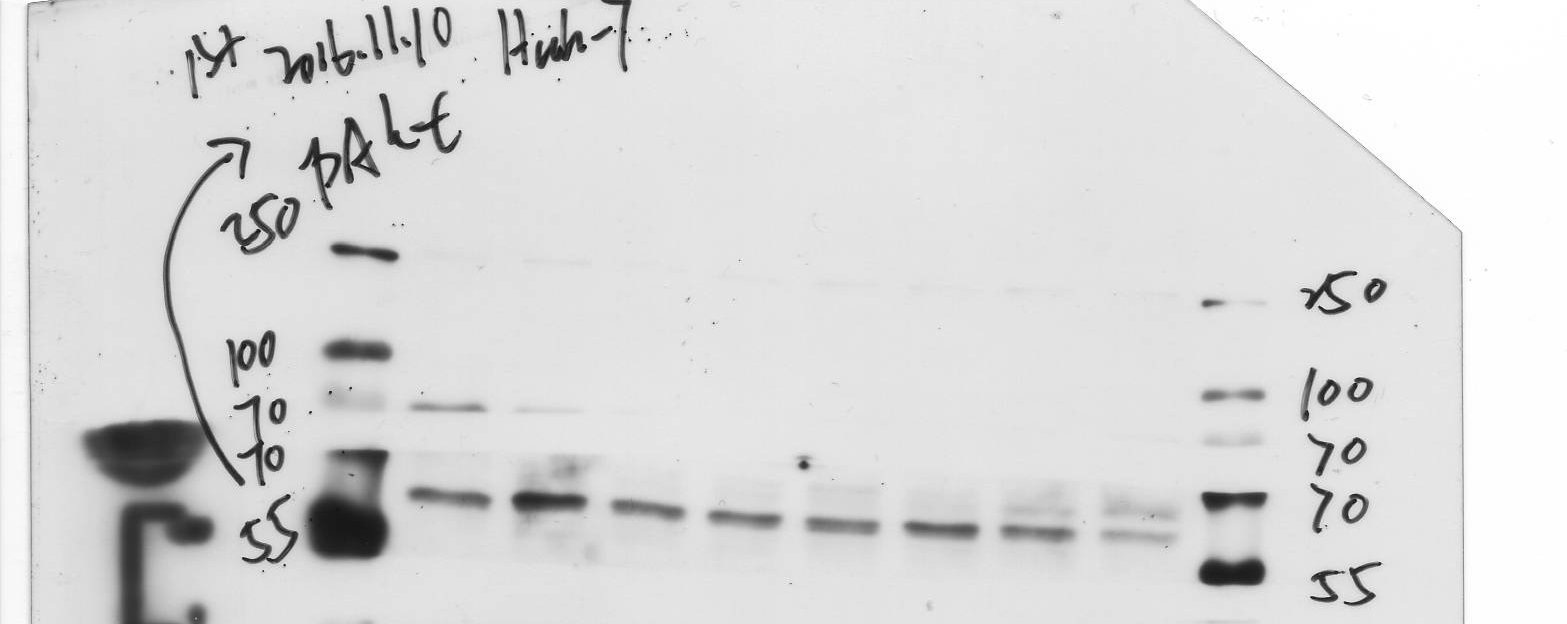

Supplement: Supplementary file 1 [file cancers-12-00615-s001.zip › cancers-670532 supplementary final/Western Blot/2016-11-10 p-akt HUH-7.jpg]

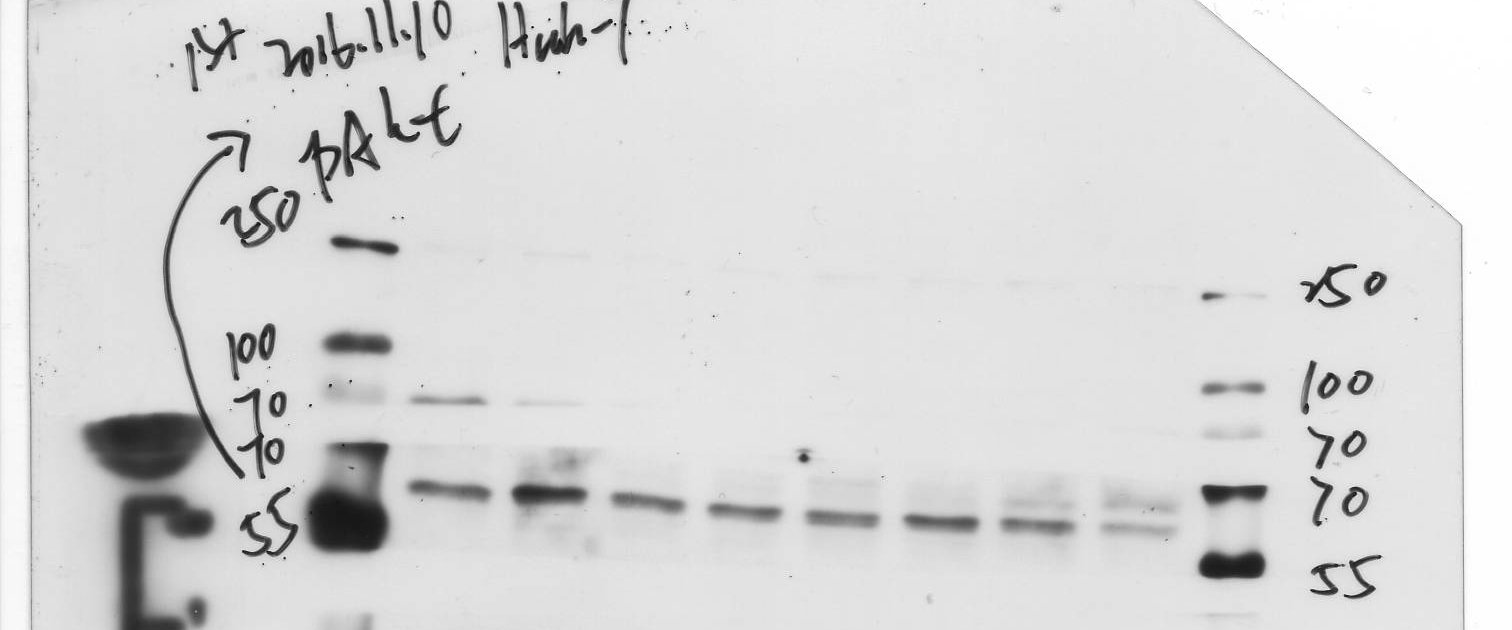

Supplement: Supplementary file 1 [file cancers-12-00615-s001.zip › cancers-670532 supplementary final/Western Blot/2016-11-10 pakt HUH-7.jpg]

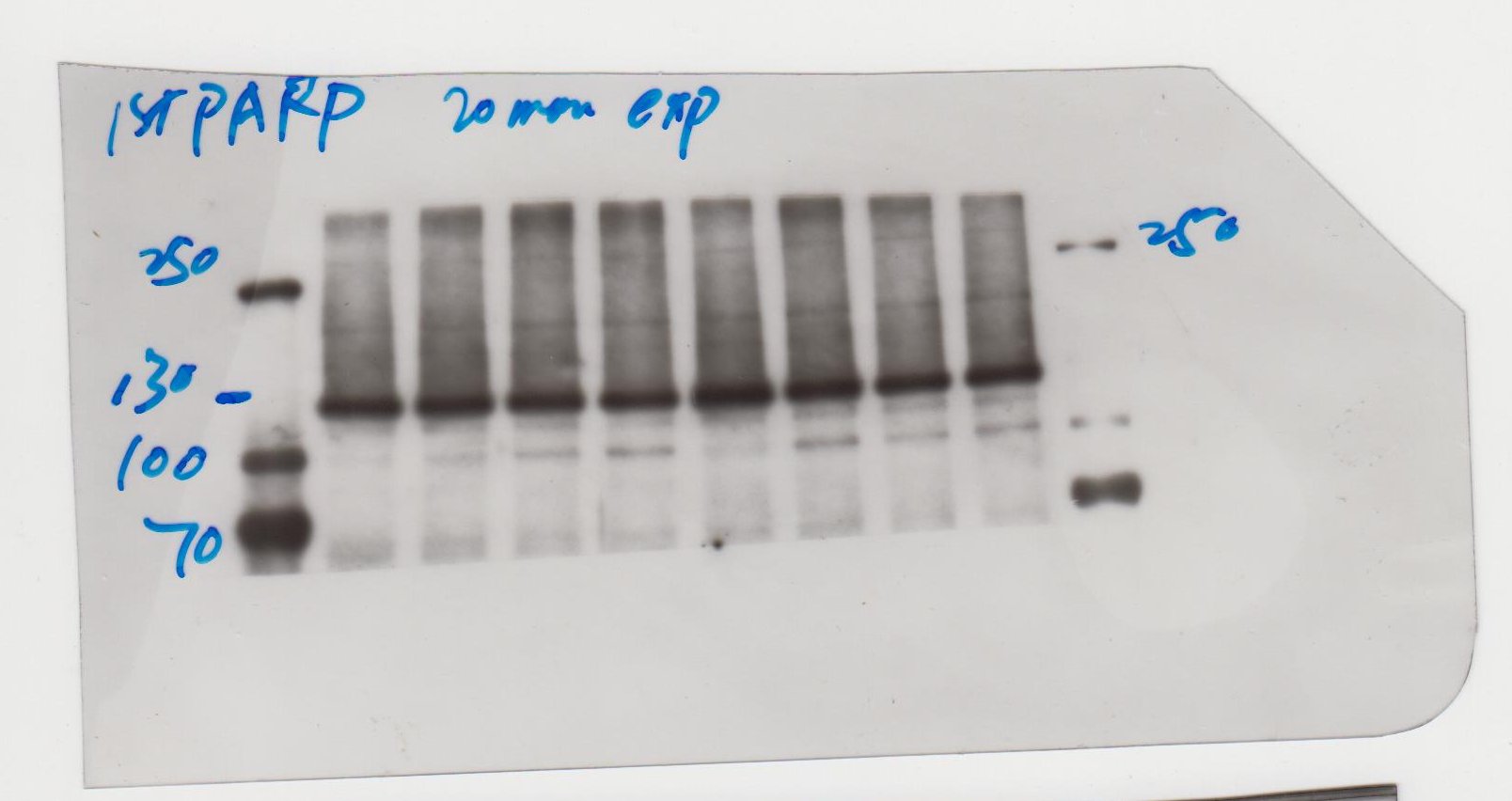

Supplement: Supplementary file 1 [file cancers-12-00615-s001.zip › cancers-670532 supplementary final/Western Blot/2017-07-15 hep 3b parp+p-erk+actin.jpg]

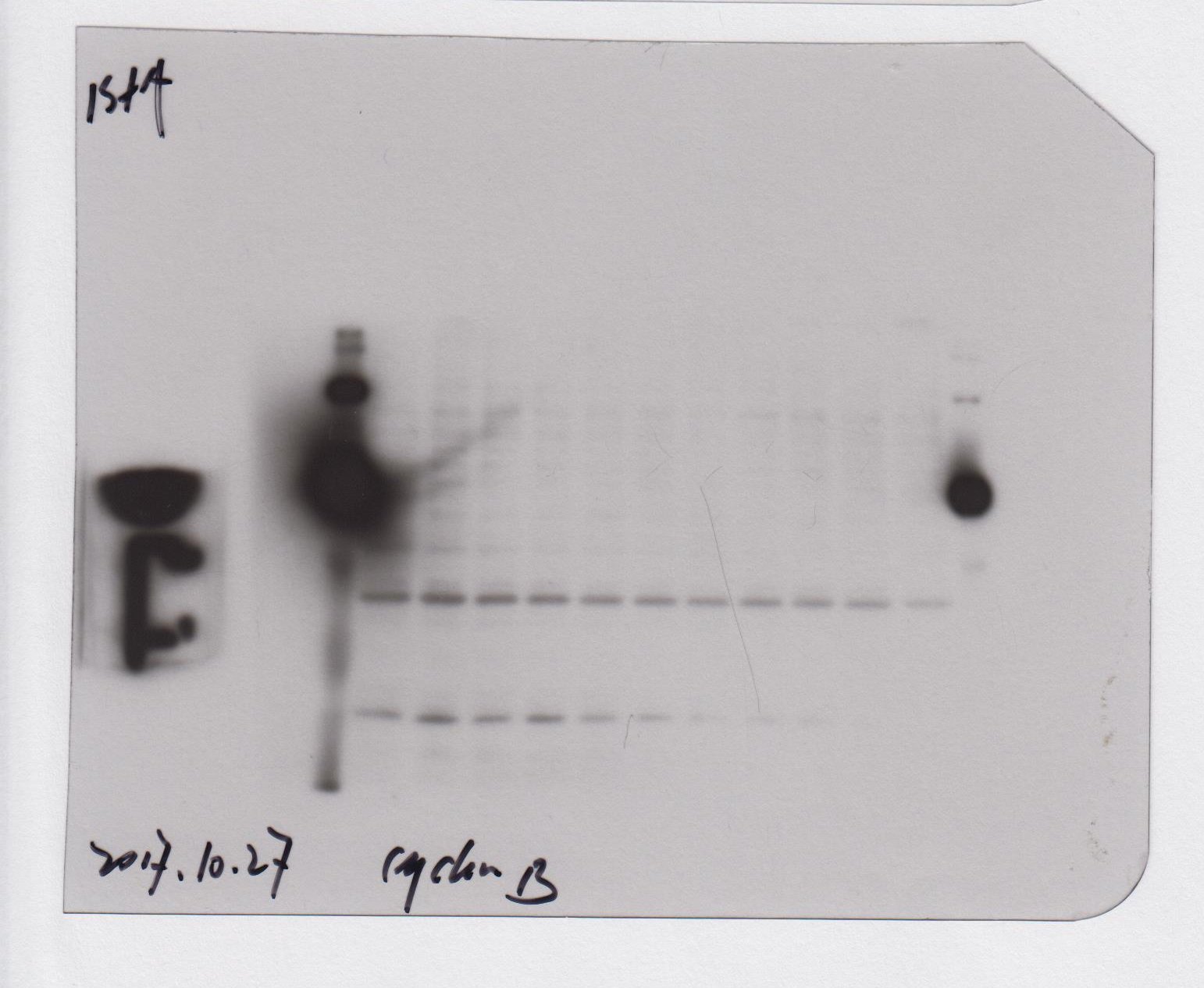

Supplement: Supplementary file 1 [file cancers-12-00615-s001.zip › cancers-670532 supplementary final/Western Blot/2017-10-27 hep 3b cyclin Bs.jpg]

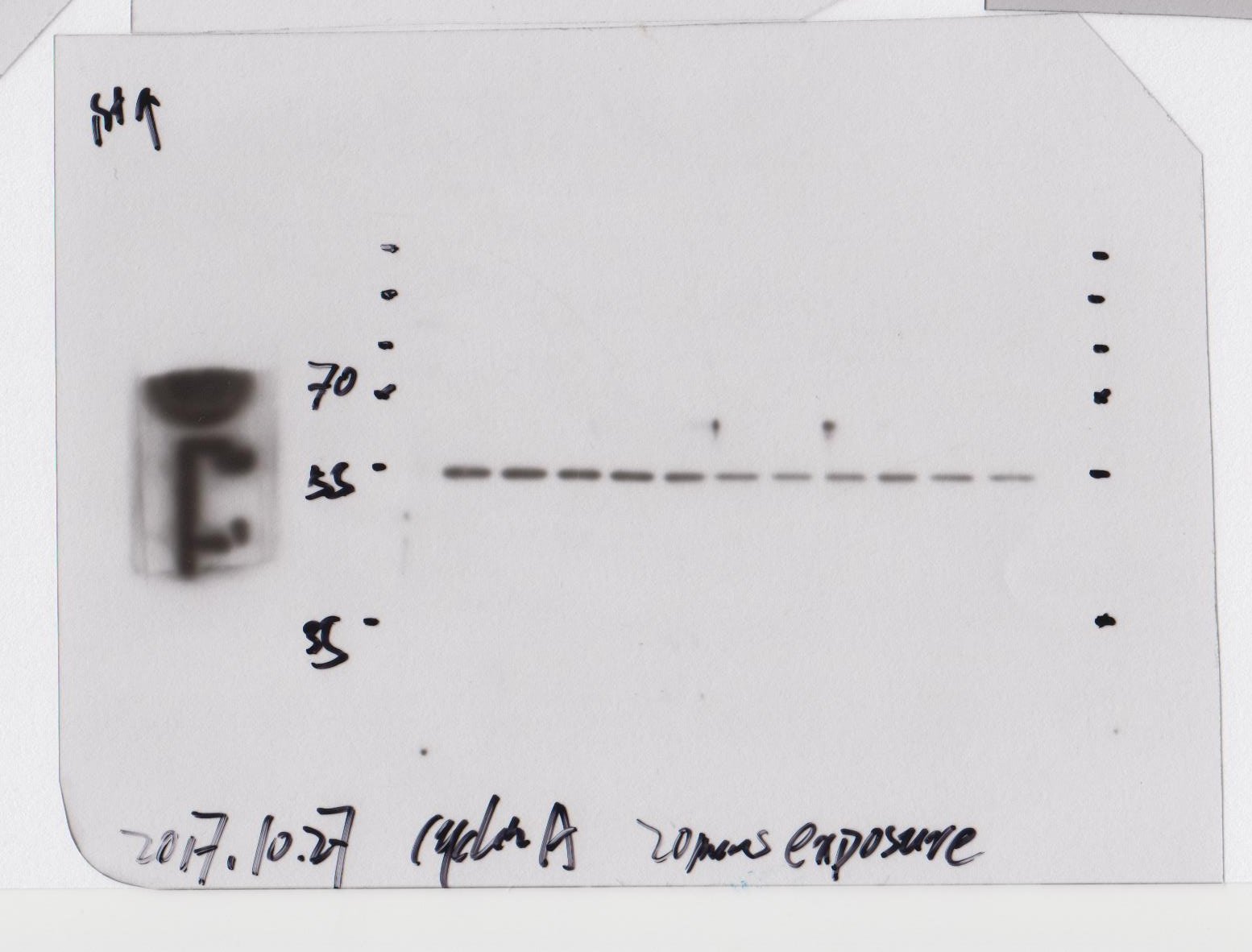

Supplement: Supplementary file 1 [file cancers-12-00615-s001.zip › cancers-670532 supplementary final/Western Blot/2017-10-27 hep 3b cyclinAs.jpg]

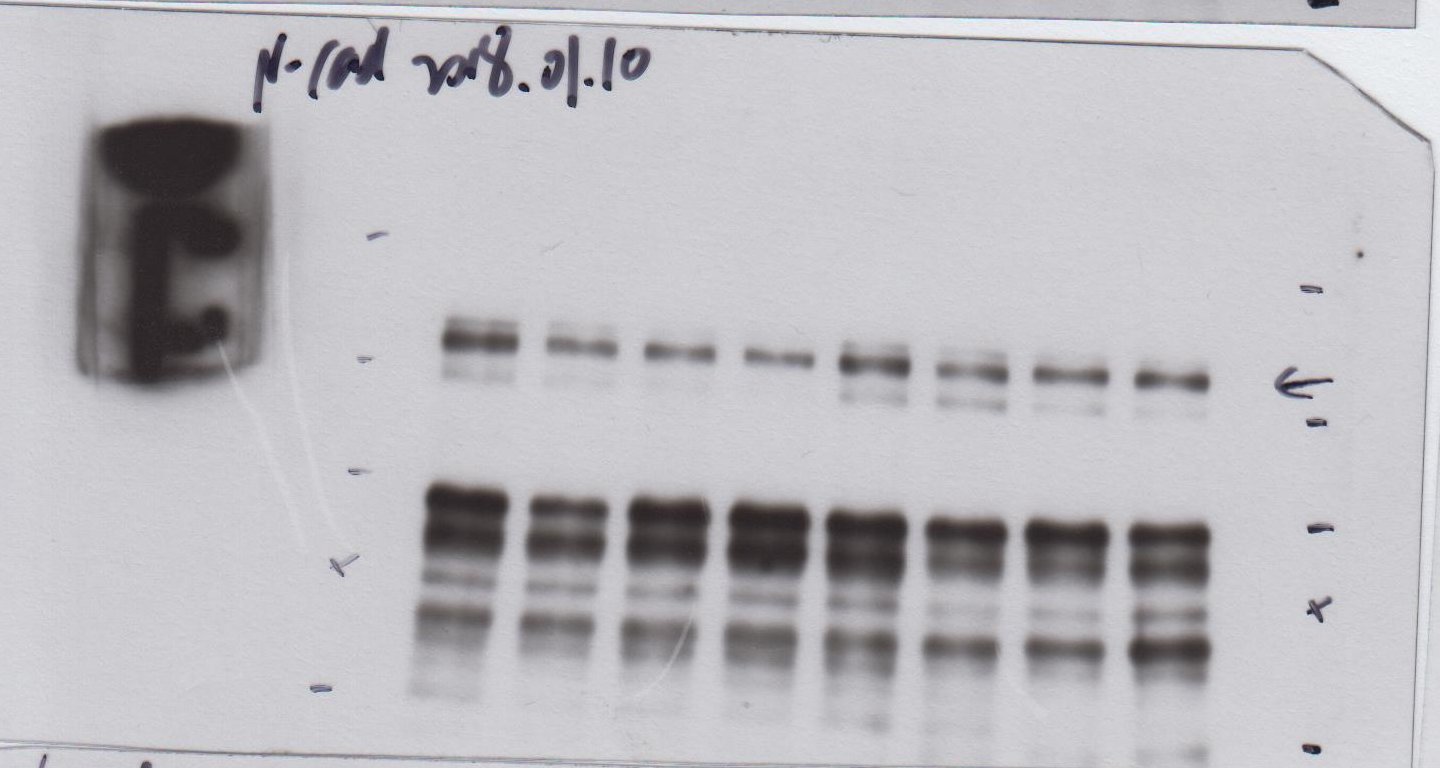

Supplement: Supplementary file 1 [file cancers-12-00615-s001.zip › cancers-670532 supplementary final/Western Blot/2018-01-10 hep 3b n-cads .jpg]

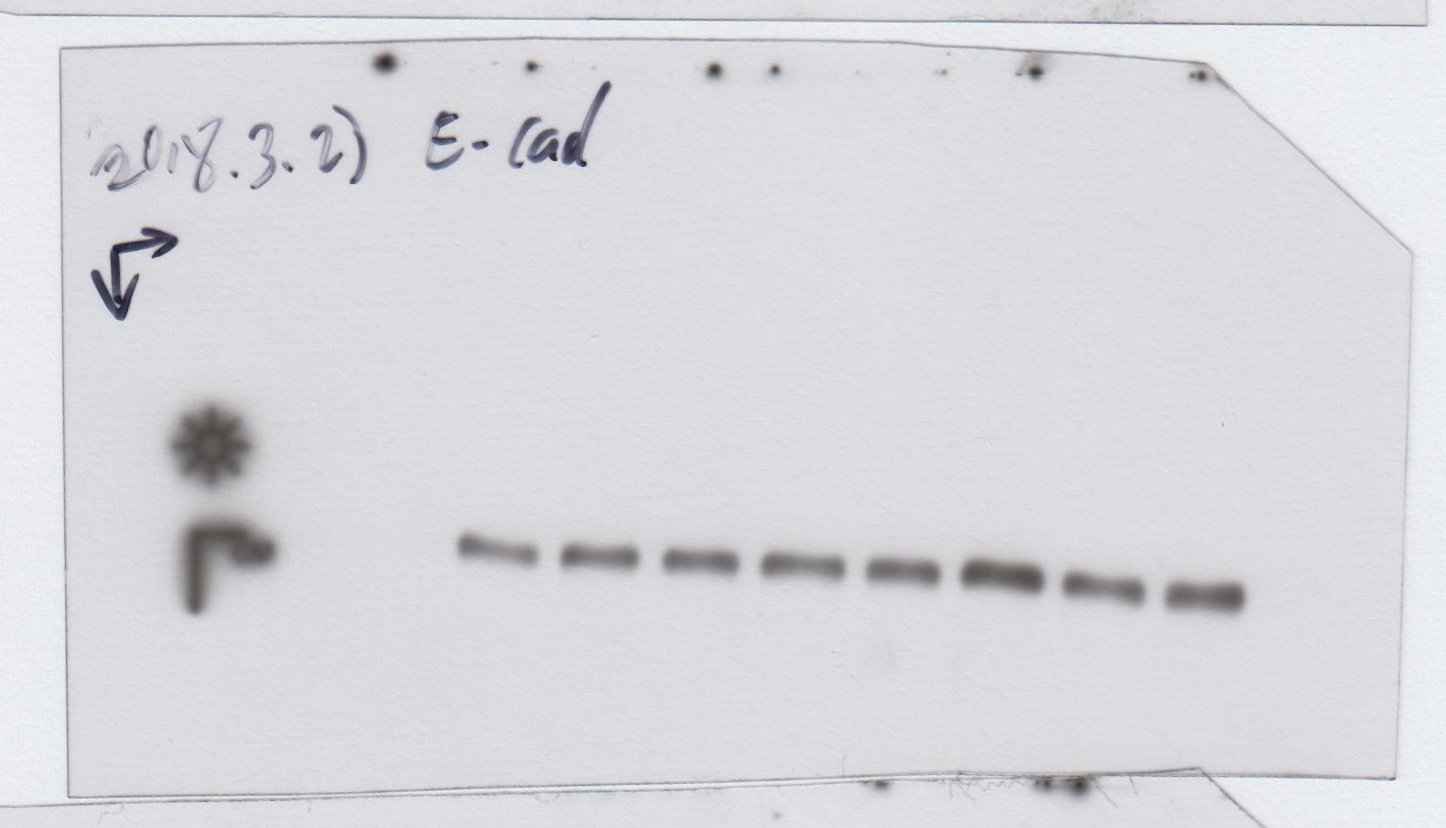

Supplement: Supplementary file 1 [file cancers-12-00615-s001.zip › cancers-670532 supplementary final/Western Blot/2018-03-23 hep 3b e-cads-2.jpg]

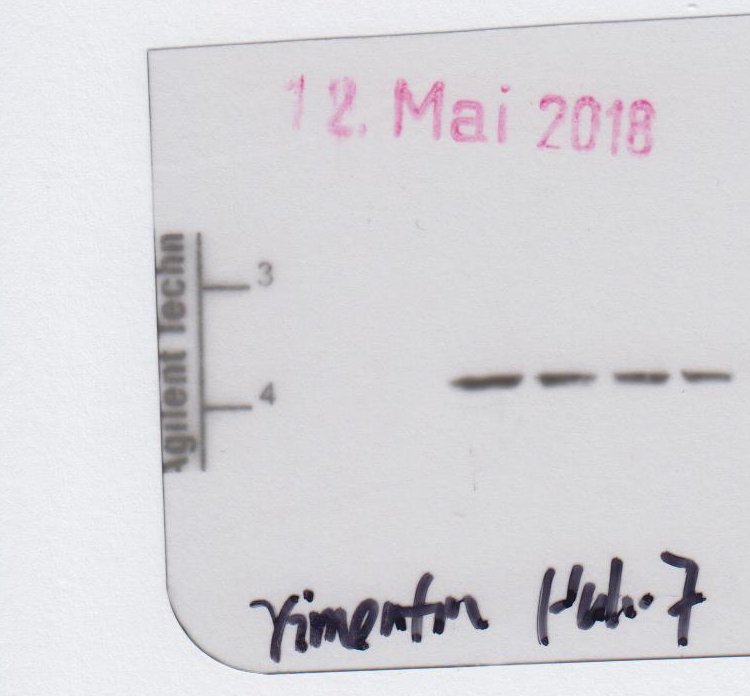

Supplement: Supplementary file 1 [file cancers-12-00615-s001.zip › cancers-670532 supplementary final/Western Blot/2018-05-12 huh -7 vimentins.jpg]

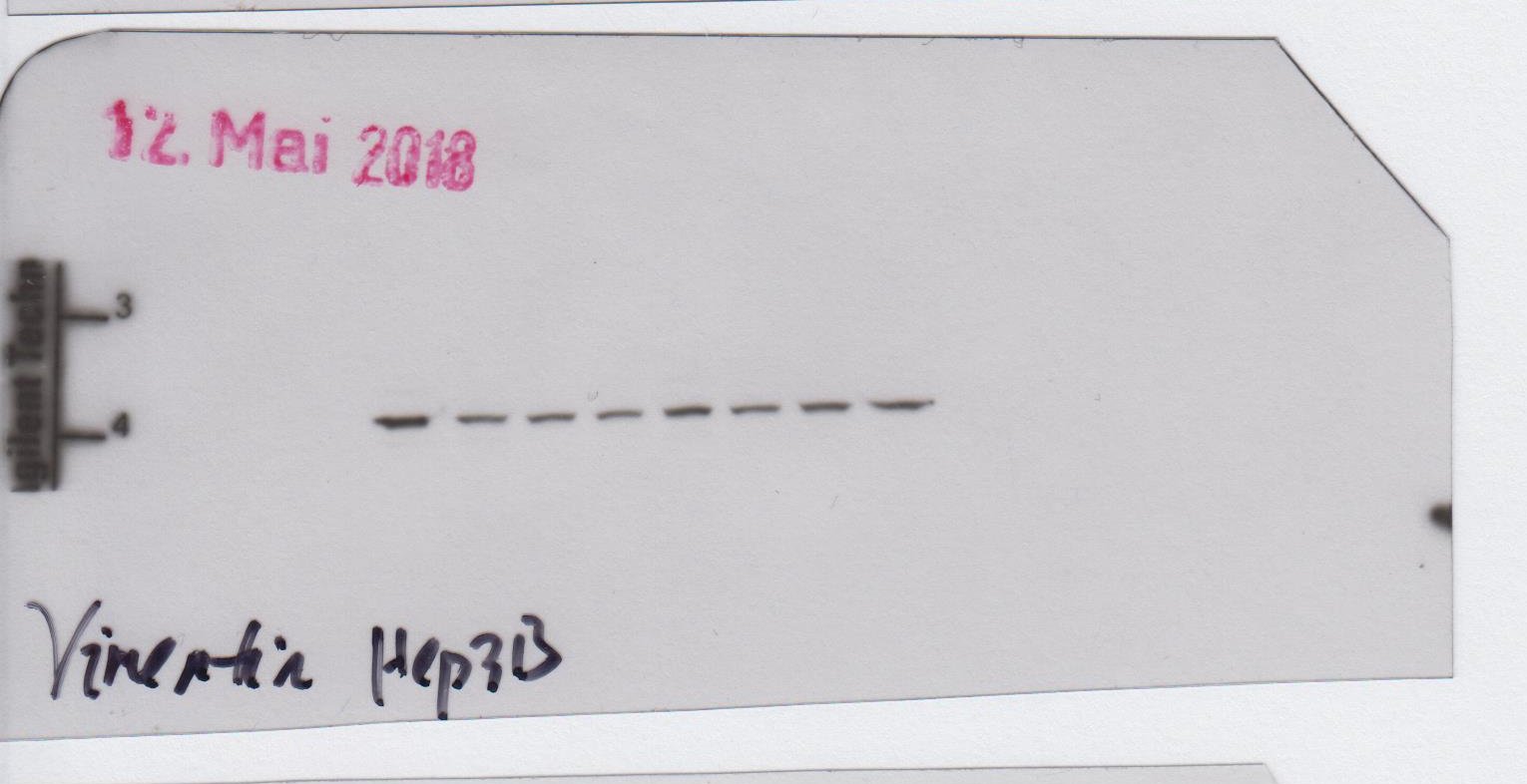

Supplement: Supplementary file 1 [file cancers-12-00615-s001.zip › cancers-670532 supplementary final/Western Blot/2018-05-12 hep 3b vimentins.jpg]

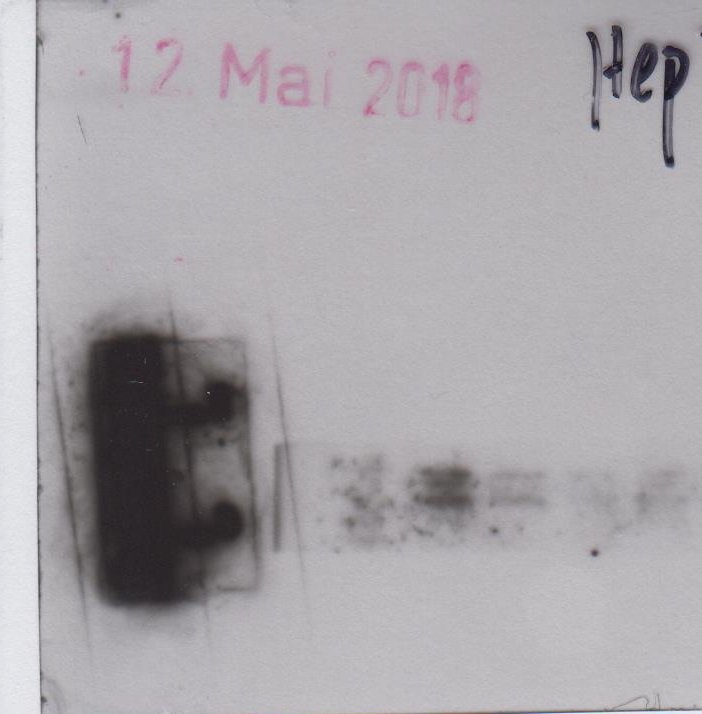

Supplement: Supplementary file 1 [file cancers-12-00615-s001.zip › cancers-670532 supplementary final/Western Blot/2018-05-12 snails hep 3b.jpg]

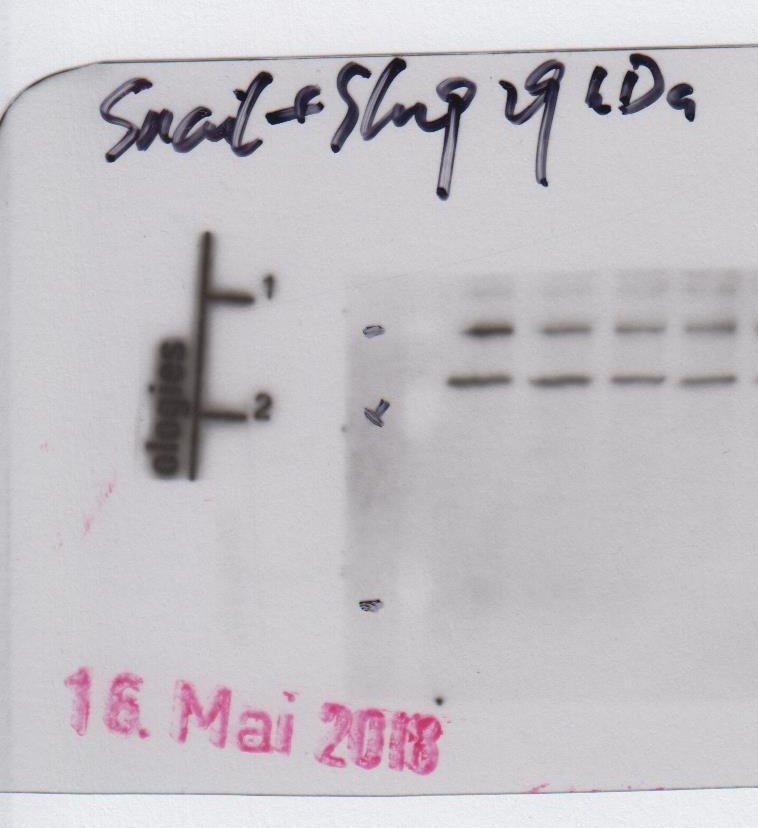

Supplement: Supplementary file 1 [file cancers-12-00615-s001.zip › cancers-670532 supplementary final/Western Blot/2018-05-16 p-akts+(snail+slug)s huh-7.jpg]

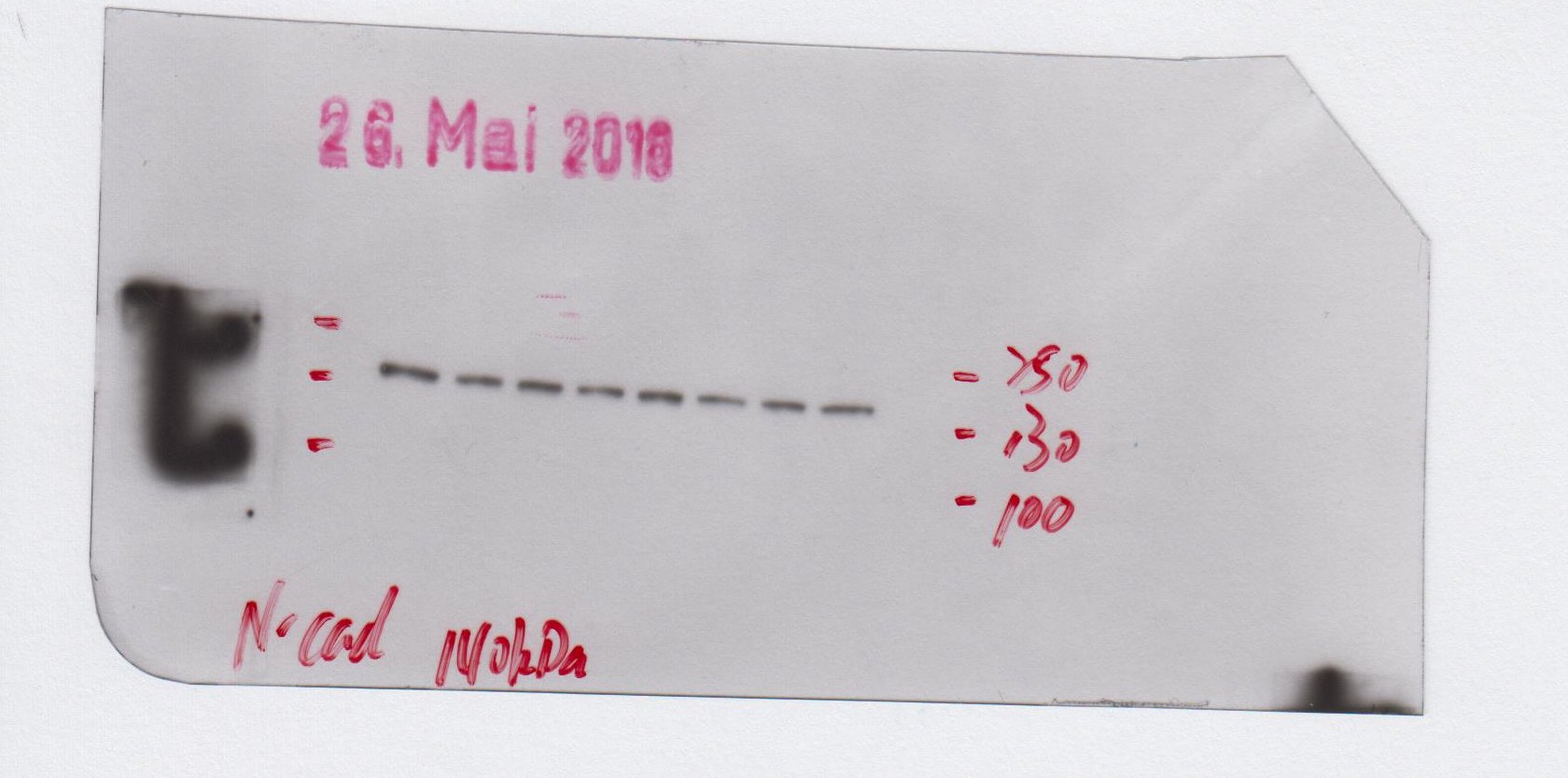

Supplement: Supplementary file 1 [file cancers-12-00615-s001.zip › cancers-670532 supplementary final/Western Blot/2018-05-26 huh-7 n-cads.jpg]

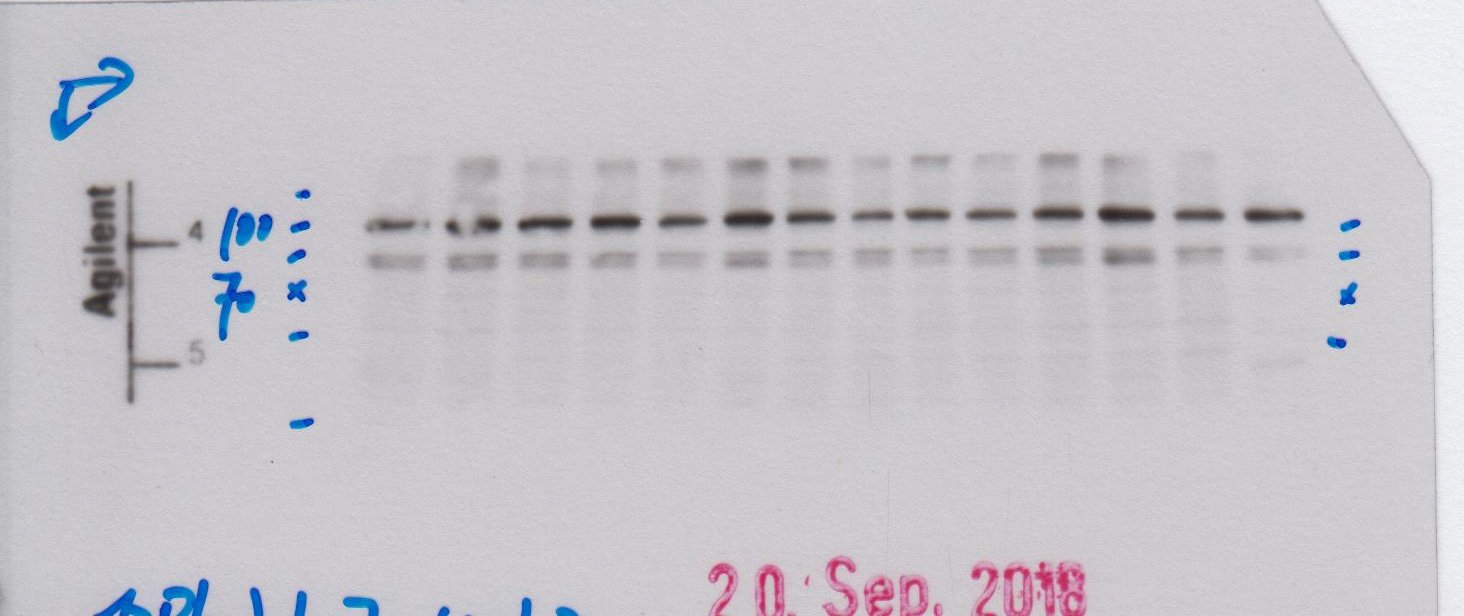

Supplement: Supplementary file 1 [file cancers-12-00615-s001.zip › cancers-670532 supplementary final/Western Blot/2018-09-19 P-RBs HUH-7.jpg]

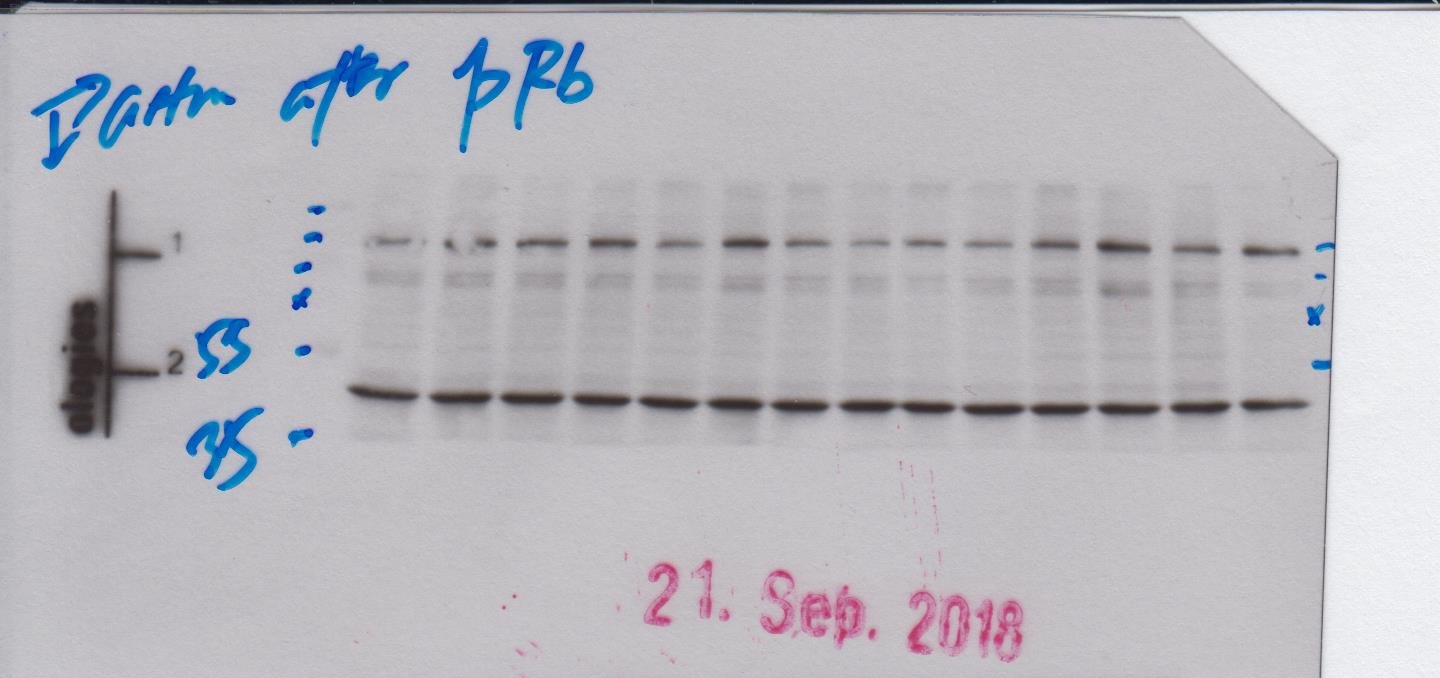

Supplement: Supplementary file 1 [file cancers-12-00615-s001.zip › cancers-670532 supplementary final/Western Blot/2018-09-21 P-RB HUH-7 .jpg]

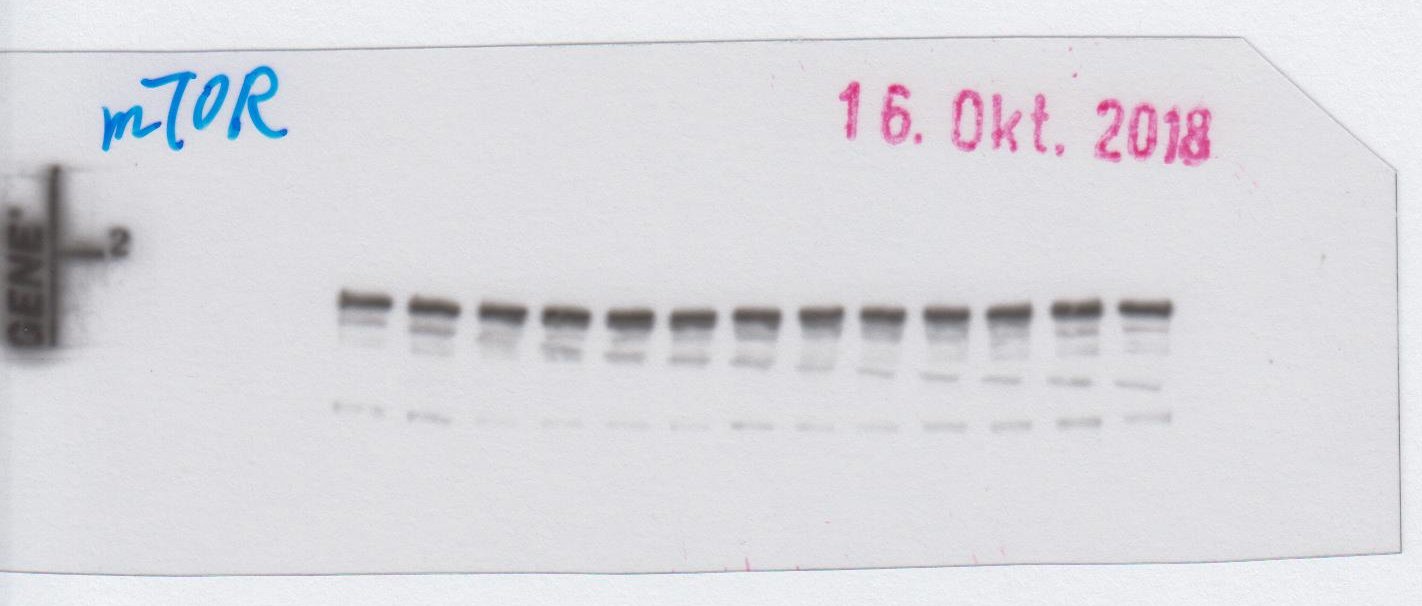

Supplement: Supplementary file 1 [file cancers-12-00615-s001.zip › cancers-670532 supplementary final/Western Blot/2018-10-16 M-TORs HUH-7.jpg]

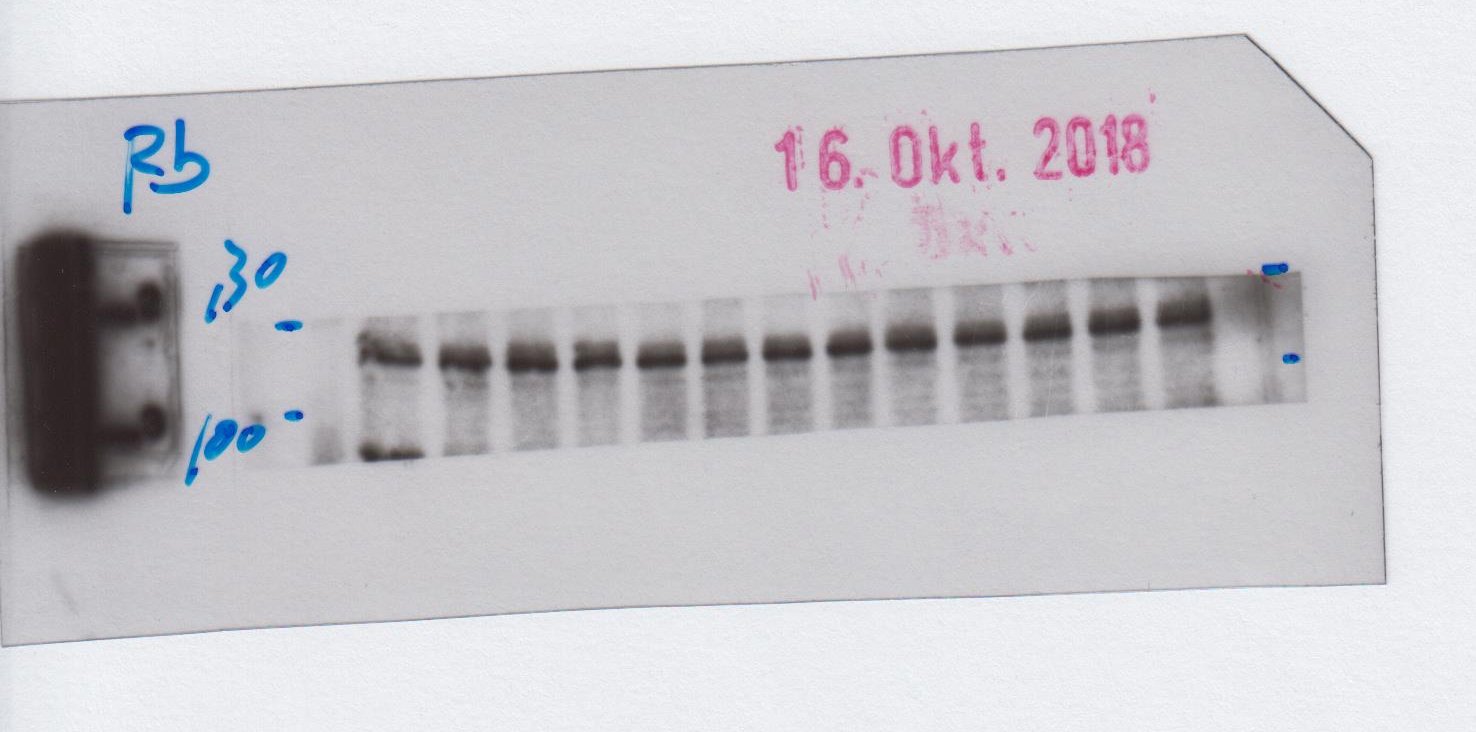

Supplement: Supplementary file 1 [file cancers-12-00615-s001.zip › cancers-670532 supplementary final/Western Blot/2018-10-16 RBs HUH-7.jpg]

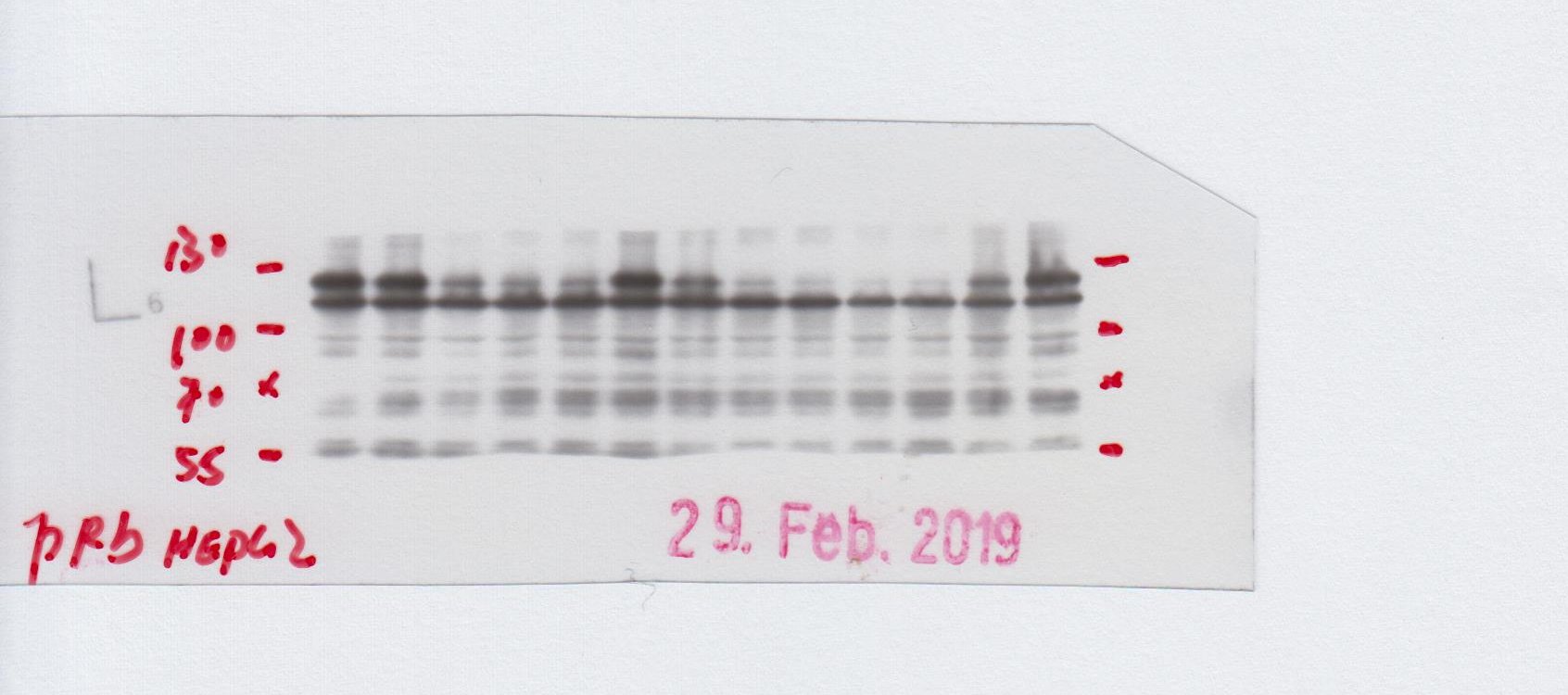

Supplement: Supplementary file 1 [file cancers-12-00615-s001.zip › cancers-670532 supplementary final/Western Blot/2019-02-19 HEP G2 p-Rbs.jpg]

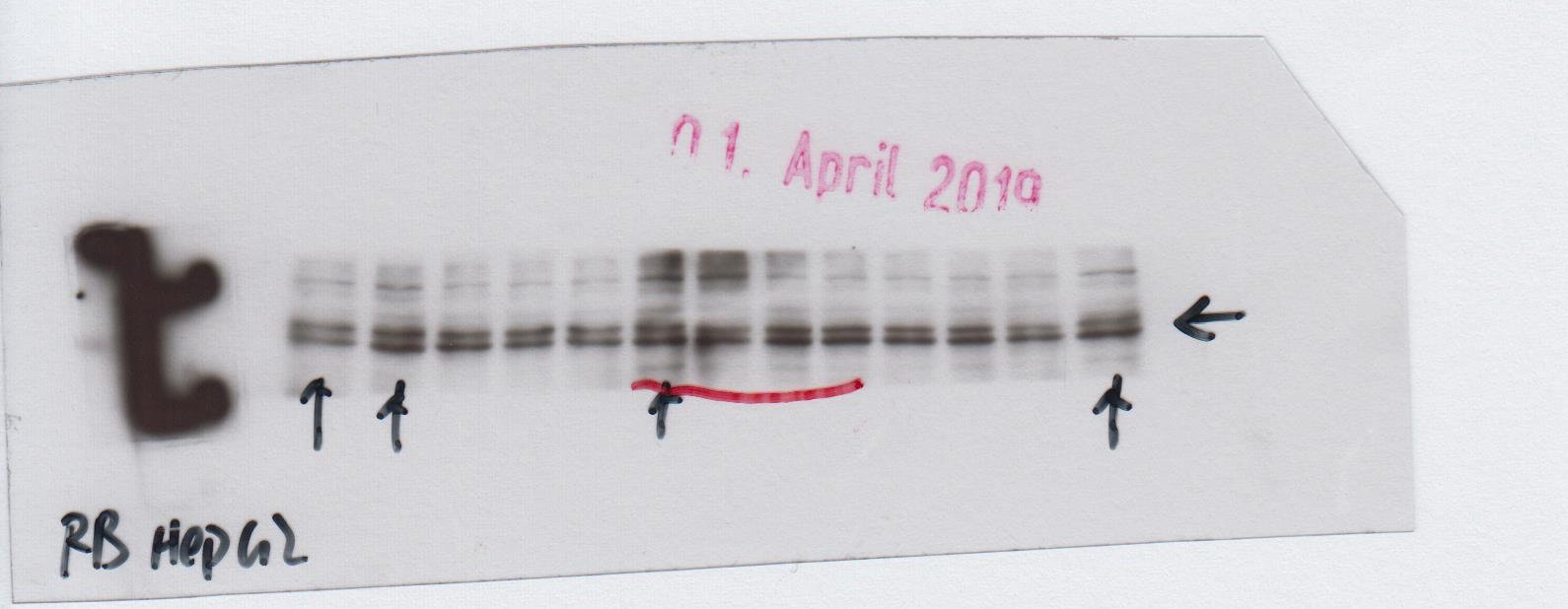

Supplement: Supplementary file 1 [file cancers-12-00615-s001.zip › cancers-670532 supplementary final/Western Blot/2019-04-01 HEP G2 RBs.jpg]

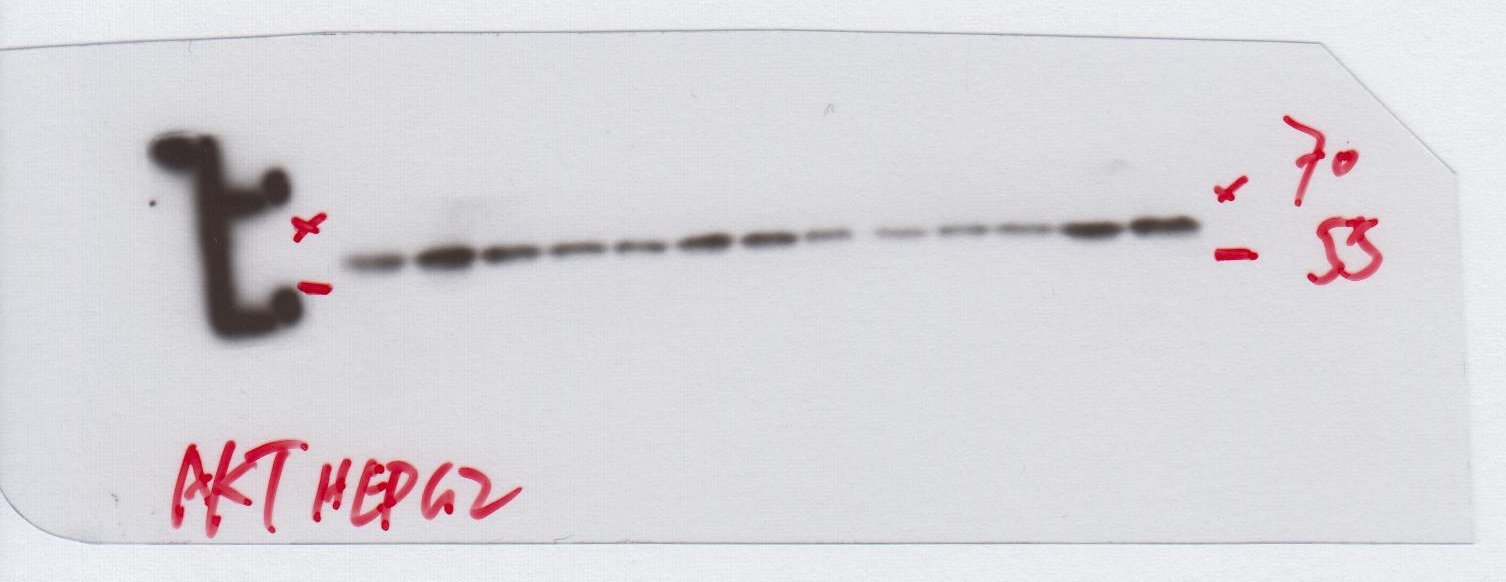

Supplement: Supplementary file 1 [file cancers-12-00615-s001.zip › cancers-670532 supplementary final/Western Blot/AKTs HEP G2.jpg]

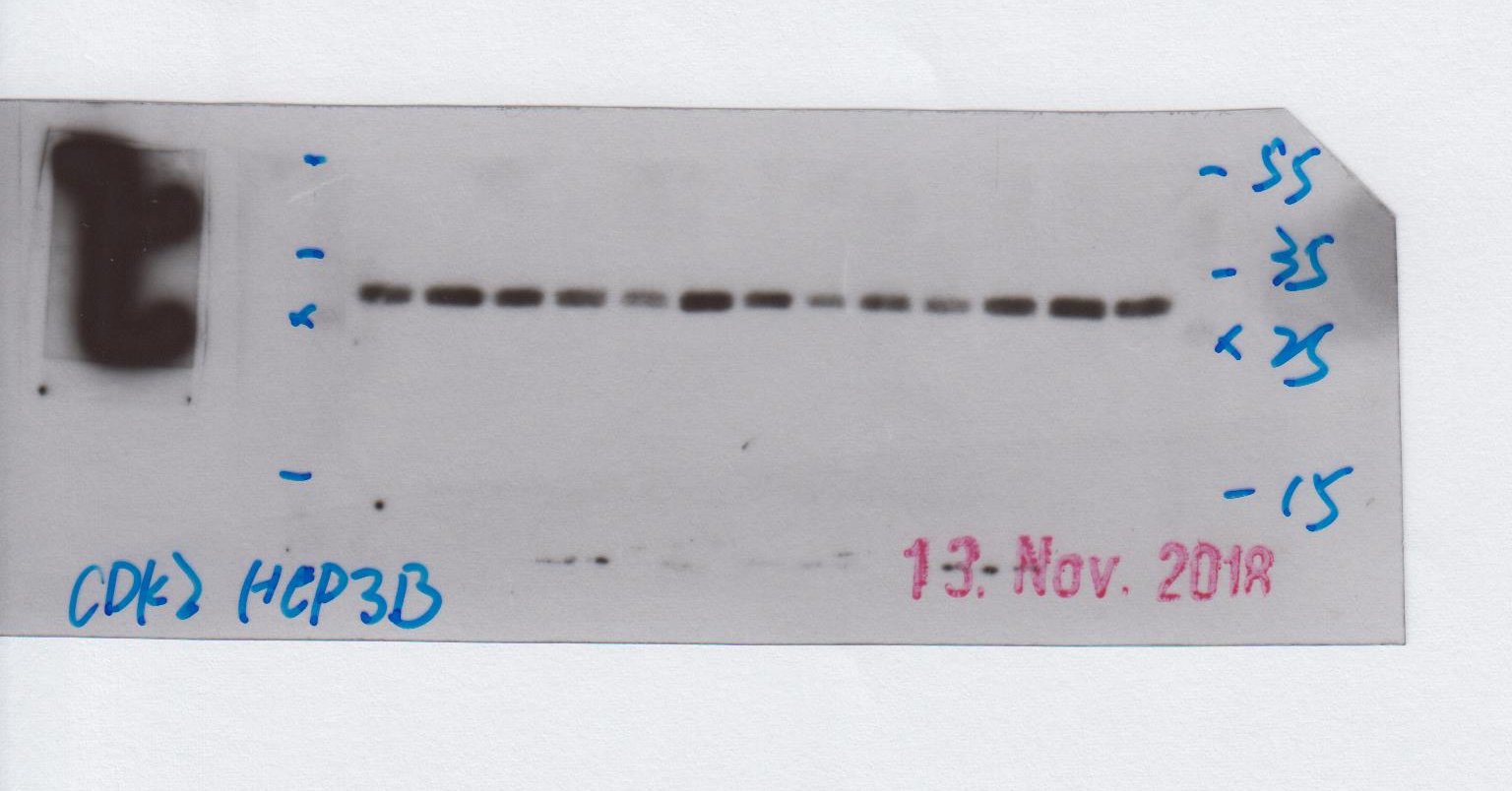

Supplement: Supplementary file 1 [file cancers-12-00615-s001.zip › cancers-670532 supplementary final/Western Blot/CDK2S hep 3b.jpg]

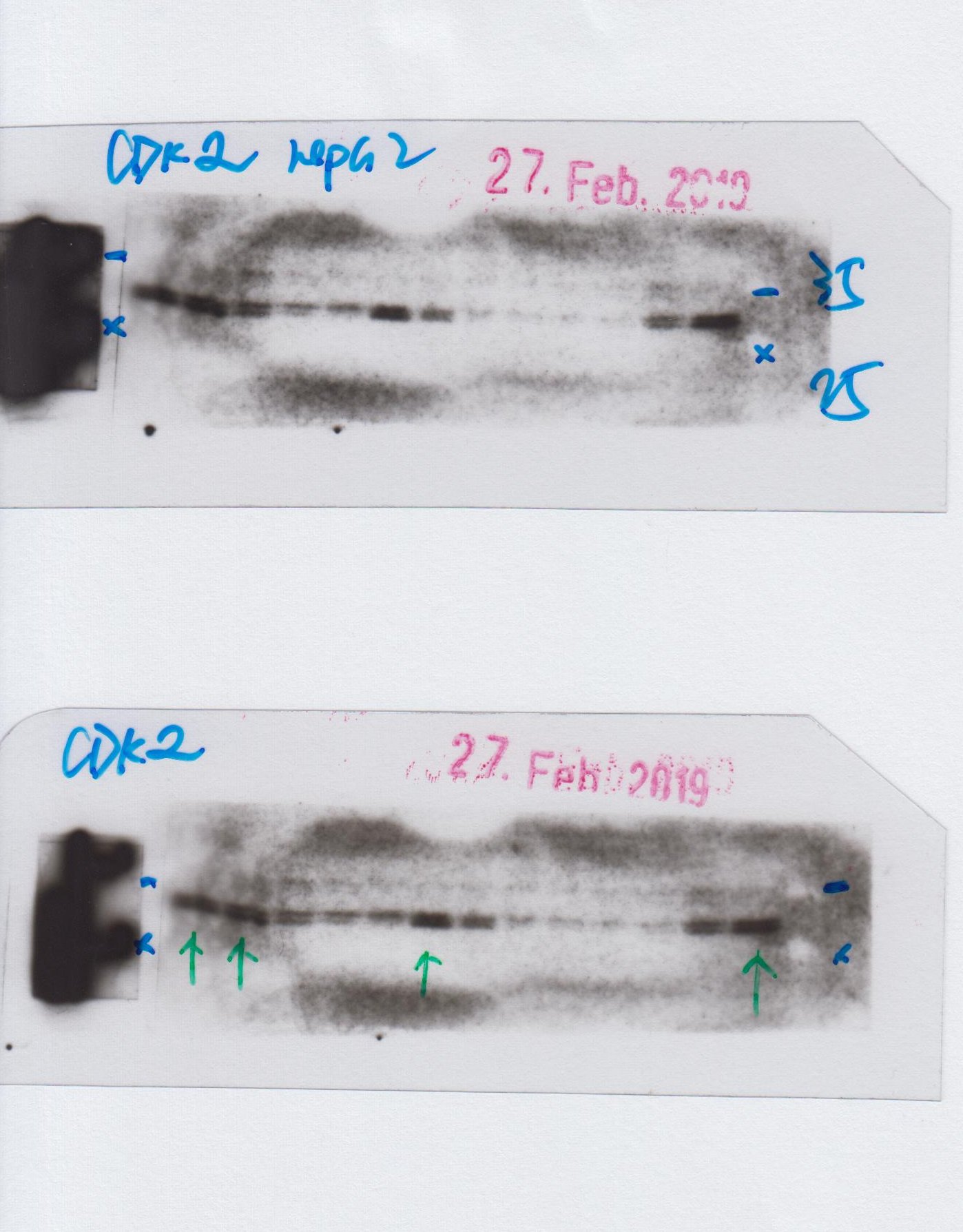

Supplement: Supplementary file 1 [file cancers-12-00615-s001.zip › cancers-670532 supplementary final/Western Blot/CDK2s hep g2╡─╕▒▒╛.jpg]

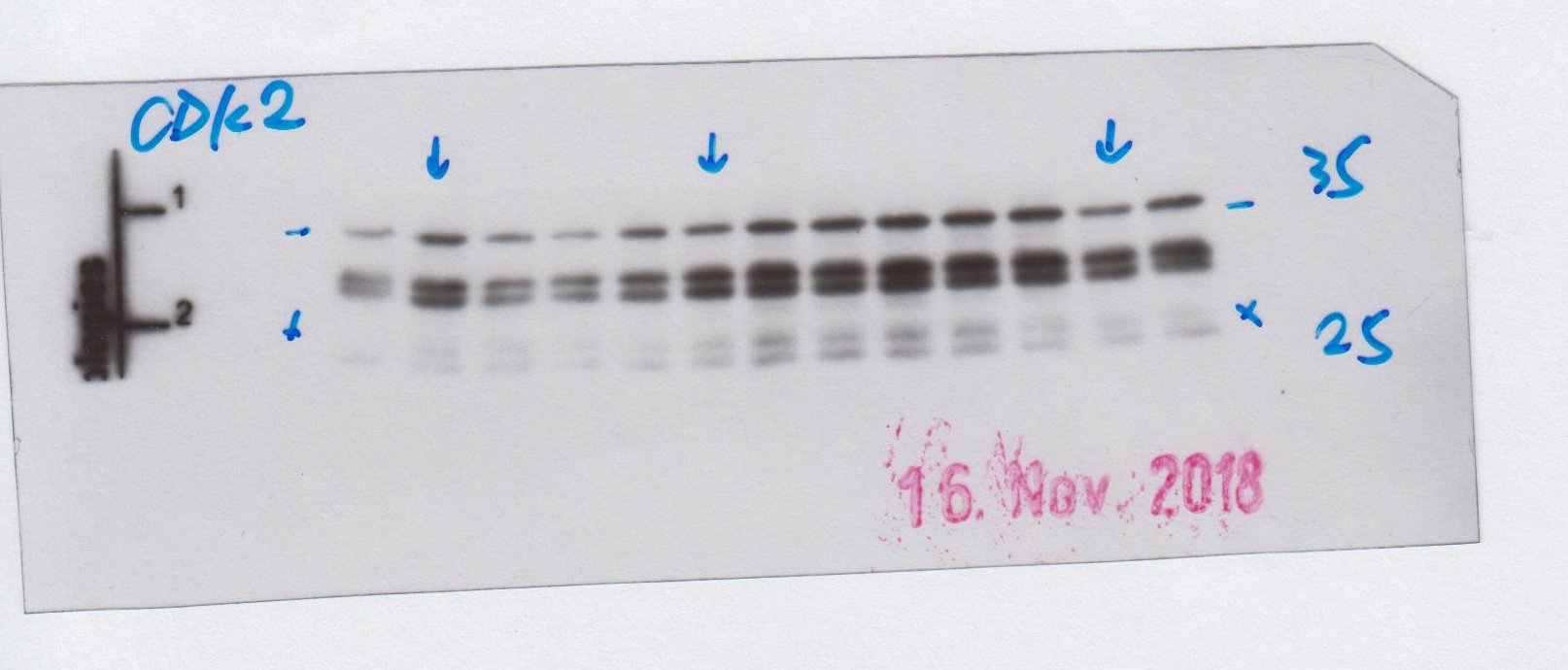

Supplement: Supplementary file 1 [file cancers-12-00615-s001.zip › cancers-670532 supplementary final/Western Blot/CDK2s HUH-7.jpg]

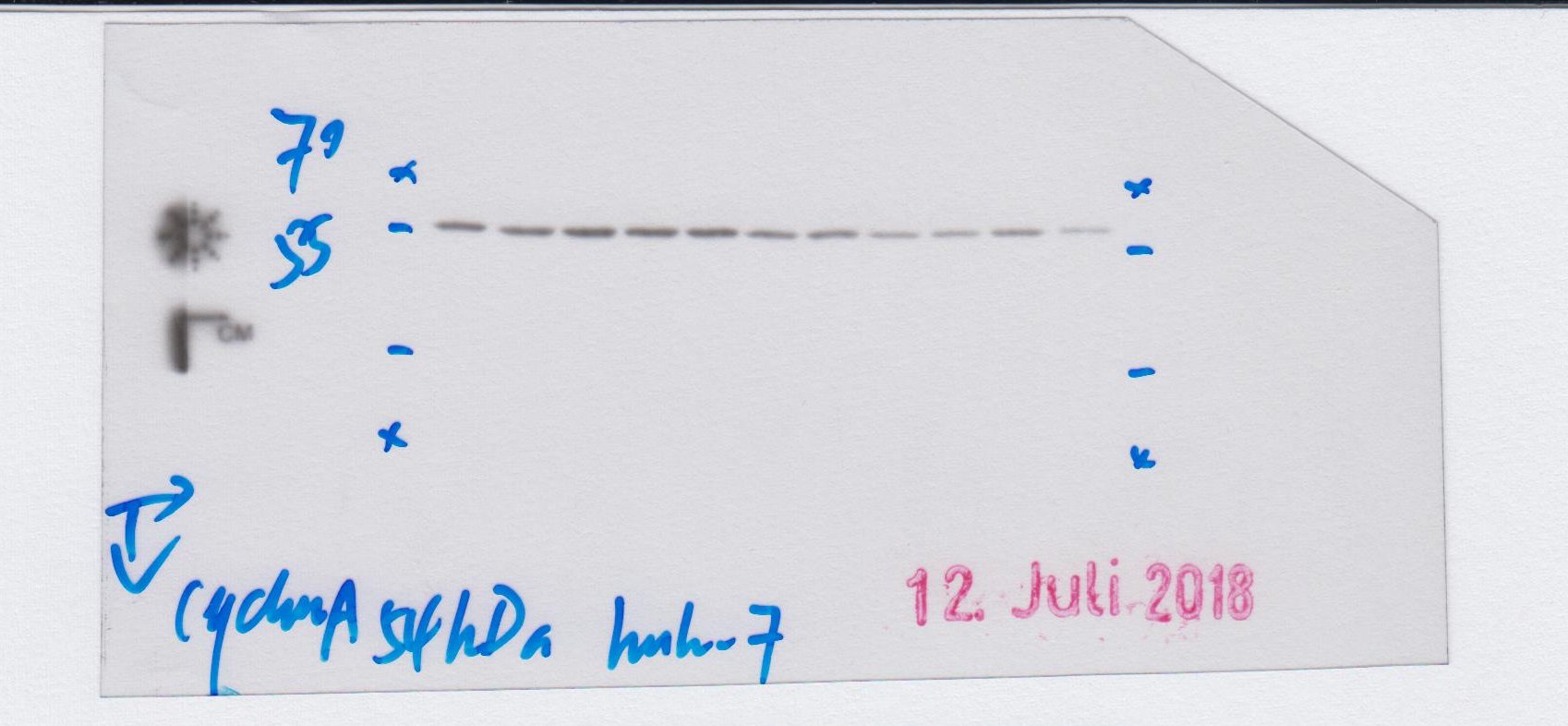

Supplement: Supplementary file 1 [file cancers-12-00615-s001.zip › cancers-670532 supplementary final/Western Blot/cyclin As kinetic huh-7.jpg]

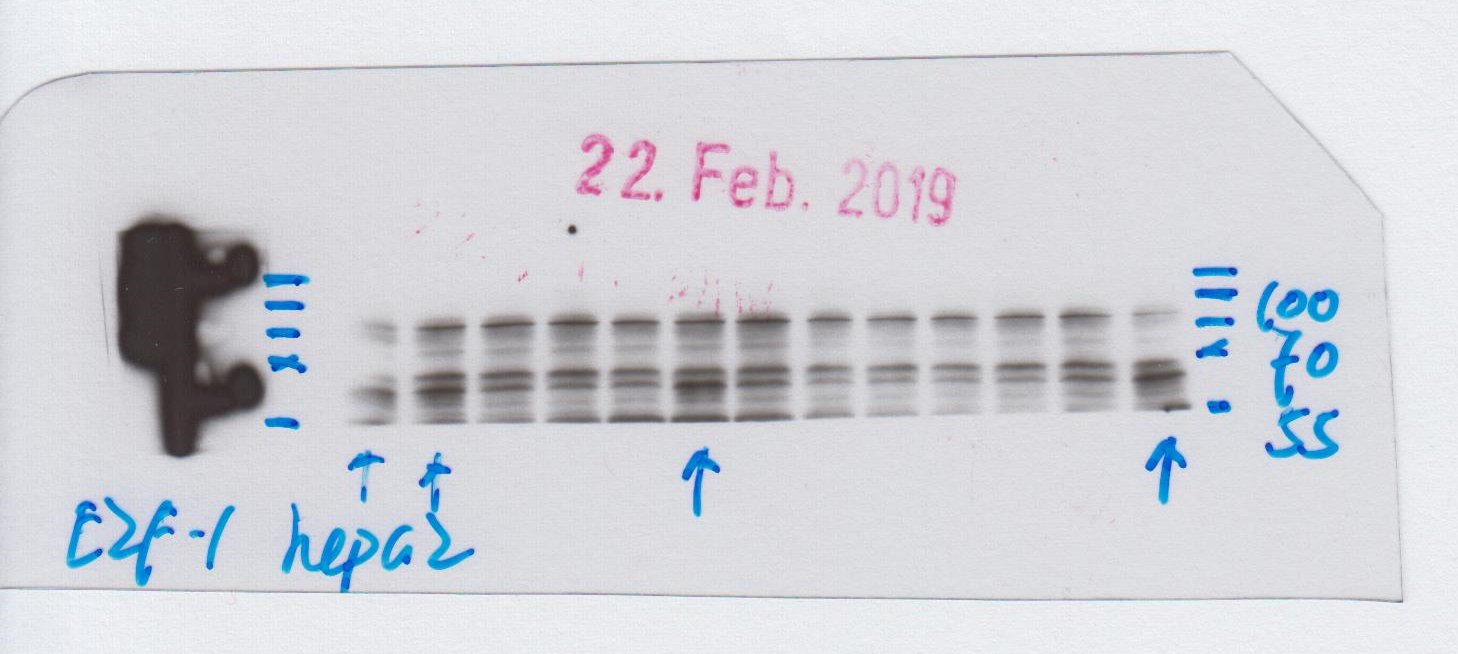

Supplement: Supplementary file 1 [file cancers-12-00615-s001.zip › cancers-670532 supplementary final/Western Blot/E2F-1 HEP G2.jpg]

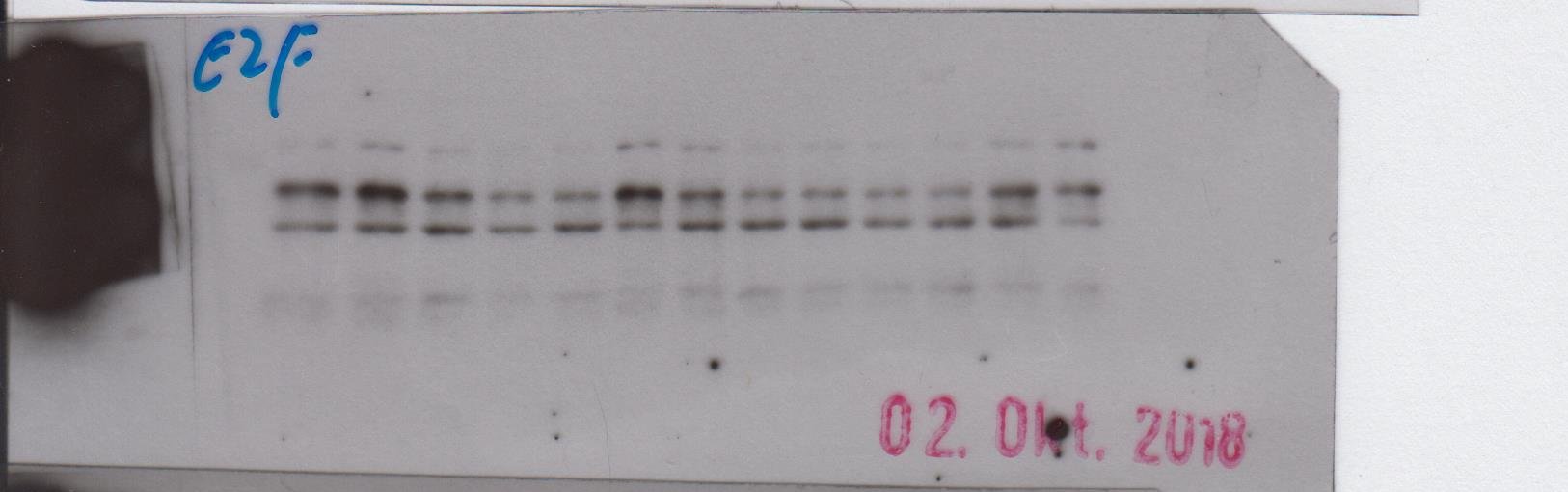

Supplement: Supplementary file 1 [file cancers-12-00615-s001.zip › cancers-670532 supplementary final/Western Blot/E2Fs HEP 3B.jpg]

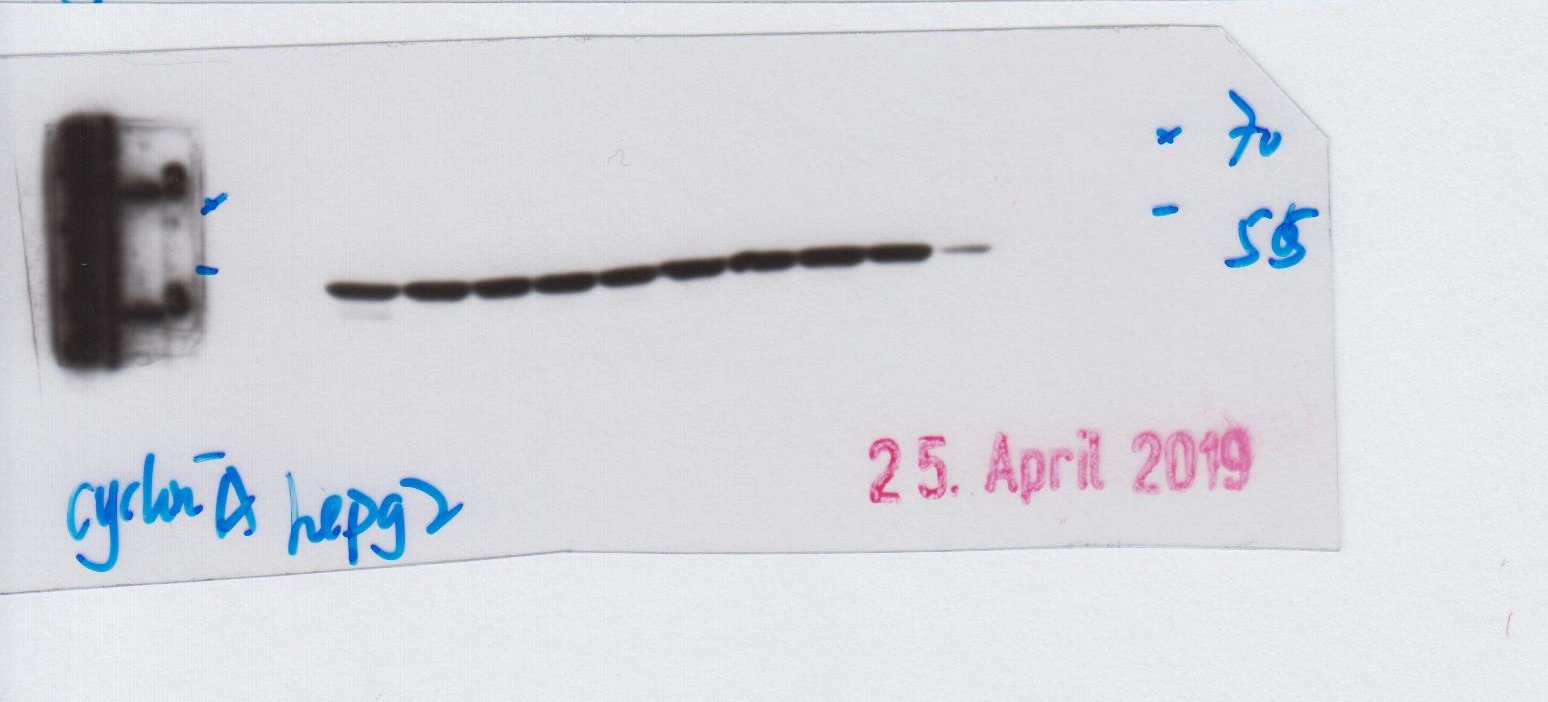

Supplement: Supplementary file 1 [file cancers-12-00615-s001.zip › cancers-670532 supplementary final/Western Blot/hep g2 cyclin As kinetic.jpg]

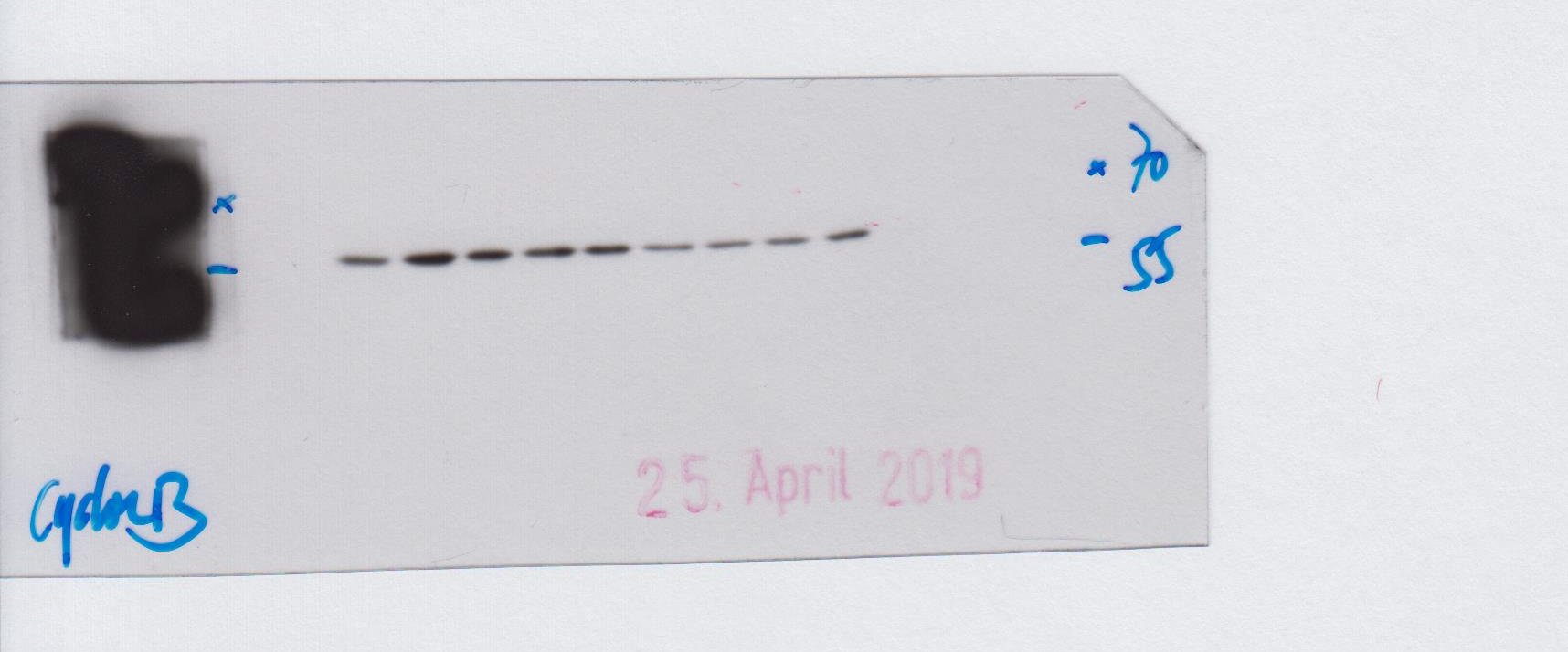

Supplement: Supplementary file 1 [file cancers-12-00615-s001.zip › cancers-670532 supplementary final/Western Blot/hep g2 cyclin Bs kinetic.jpg]

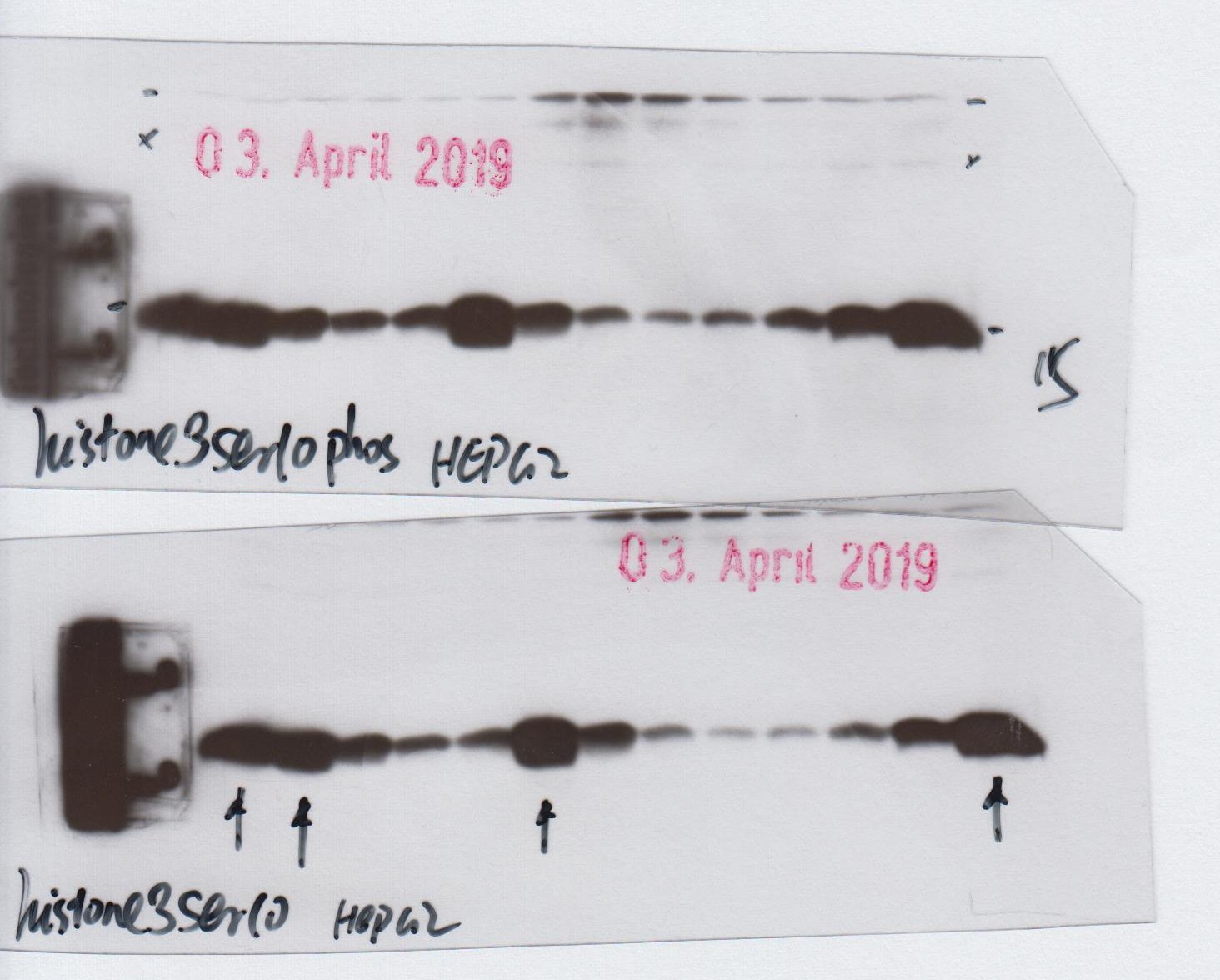

Supplement: Supplementary file 1 [file cancers-12-00615-s001.zip › cancers-670532 supplementary final/Western Blot/histone 3 ser 10 s.jpg]

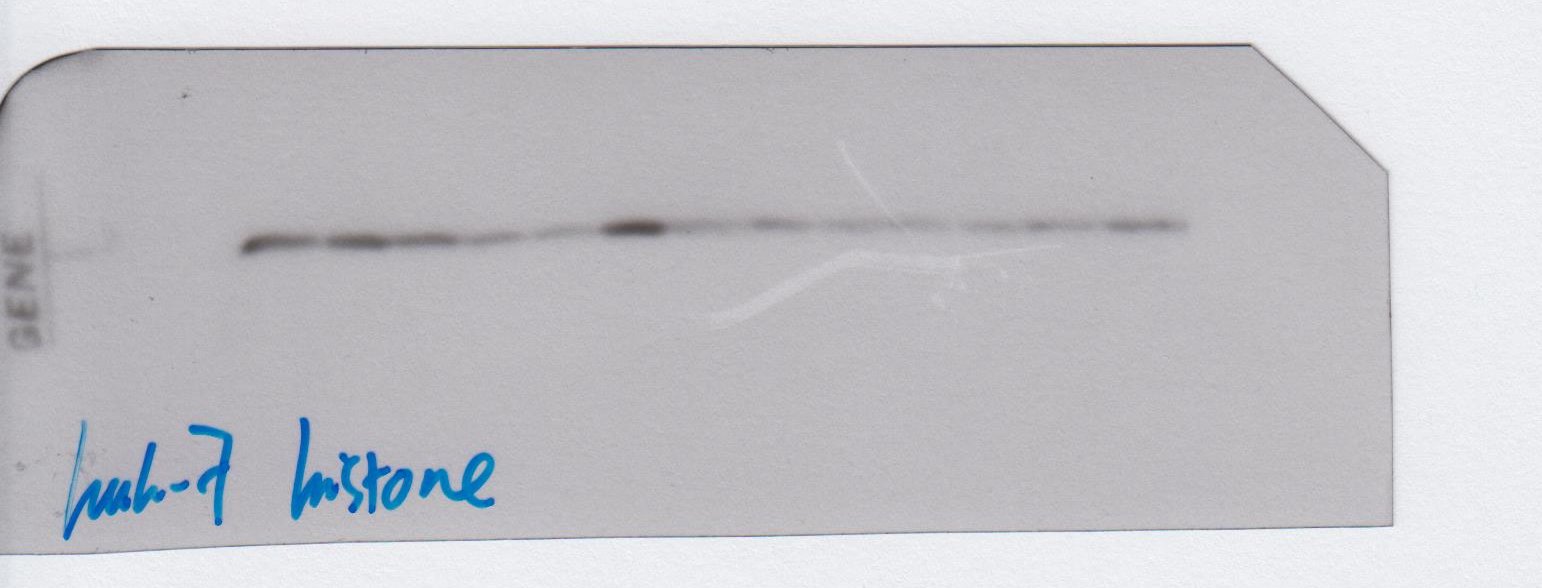

Supplement: Supplementary file 1 [file cancers-12-00615-s001.zip › cancers-670532 supplementary final/Western Blot/Histone3 P-10s Huh-7.jpg]

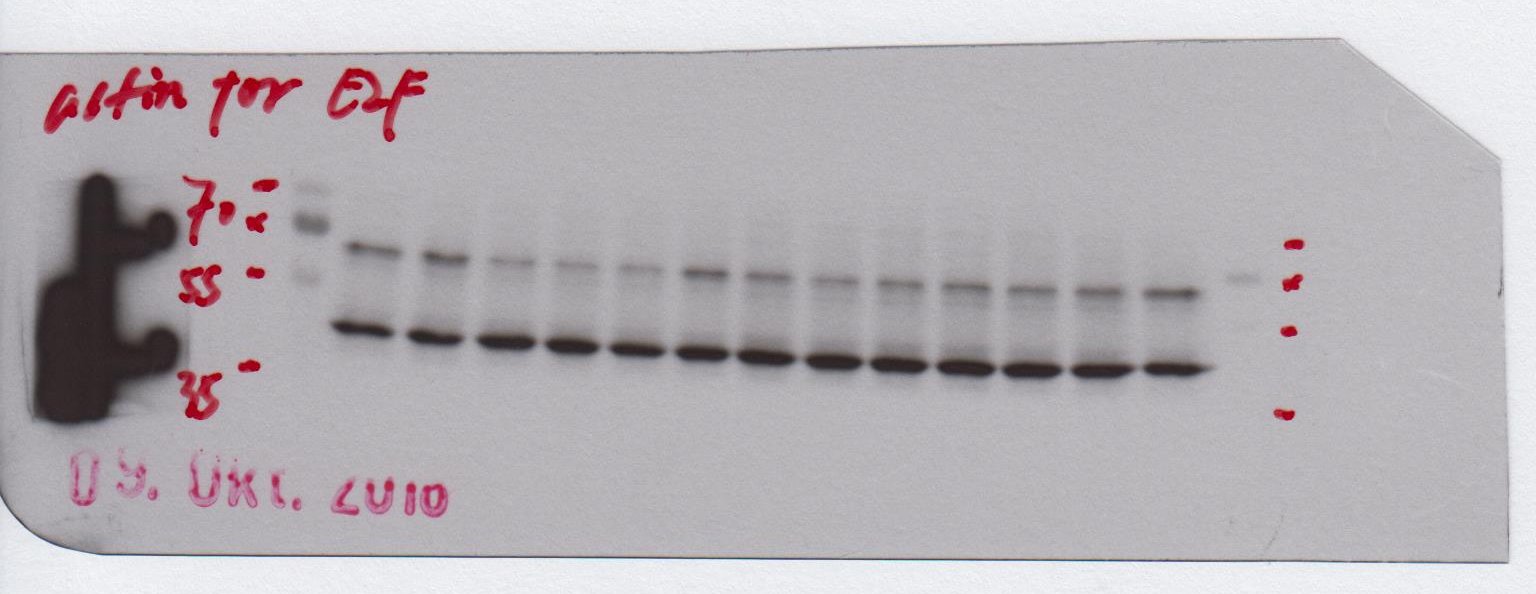

Supplement: Supplementary file 1 [file cancers-12-00615-s001.zip › cancers-670532 supplementary final/Western Blot/HUH-7 E2F-1.jpg]

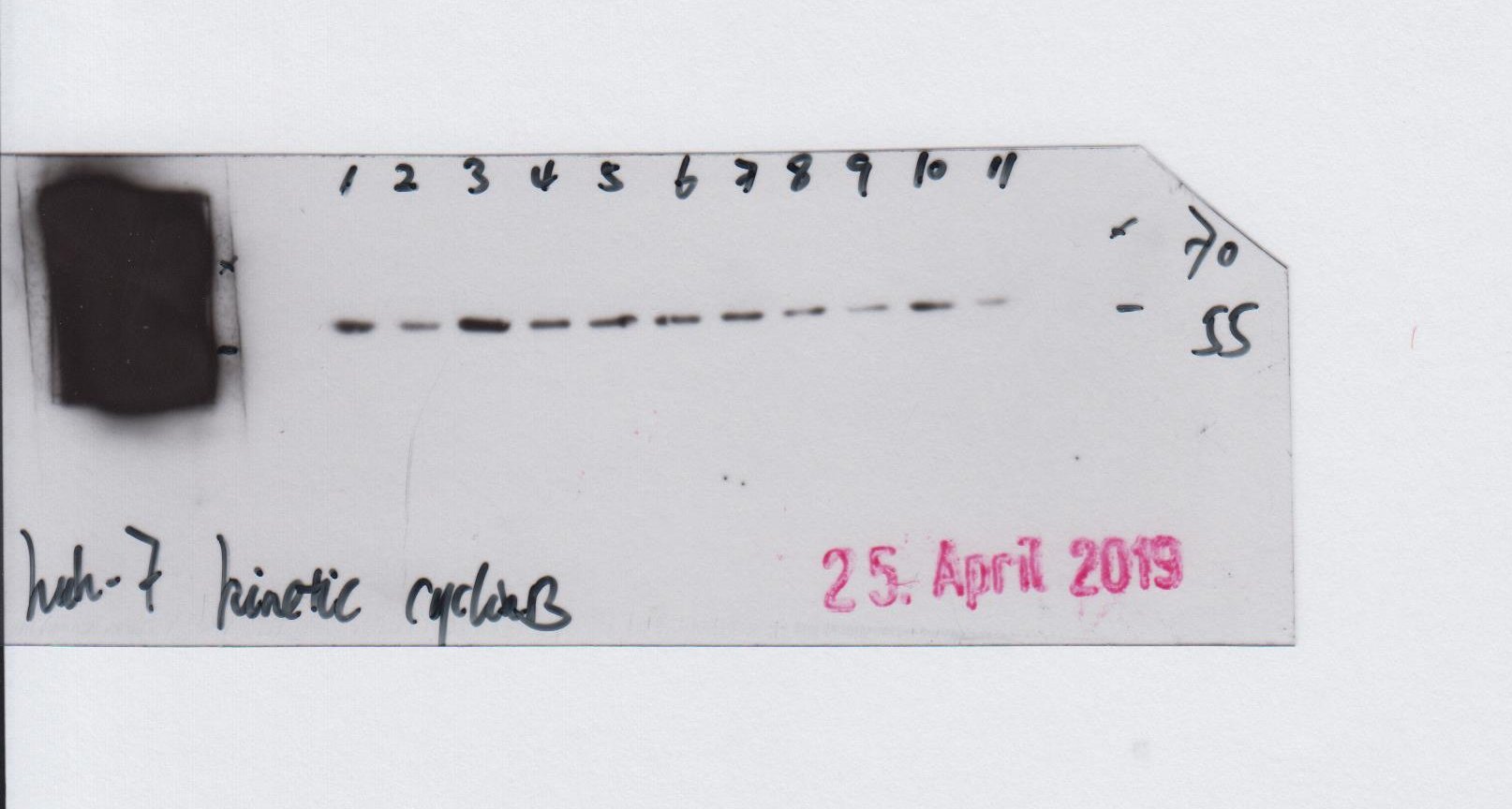

Supplement: Supplementary file 1 [file cancers-12-00615-s001.zip › cancers-670532 supplementary final/Western Blot/huh-7 kinetic cyclinBs.jpg]

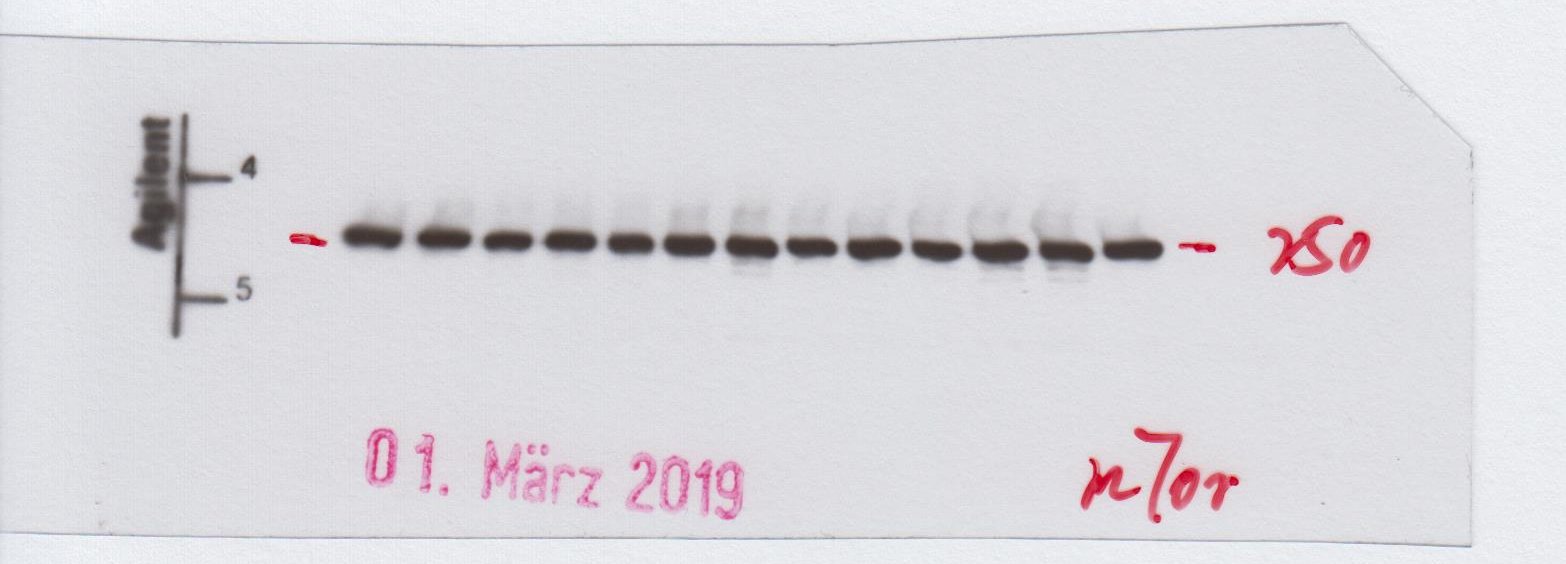

Supplement: Supplementary file 1 [file cancers-12-00615-s001.zip › cancers-670532 supplementary final/Western Blot/mTORs HEP G2.jpg]

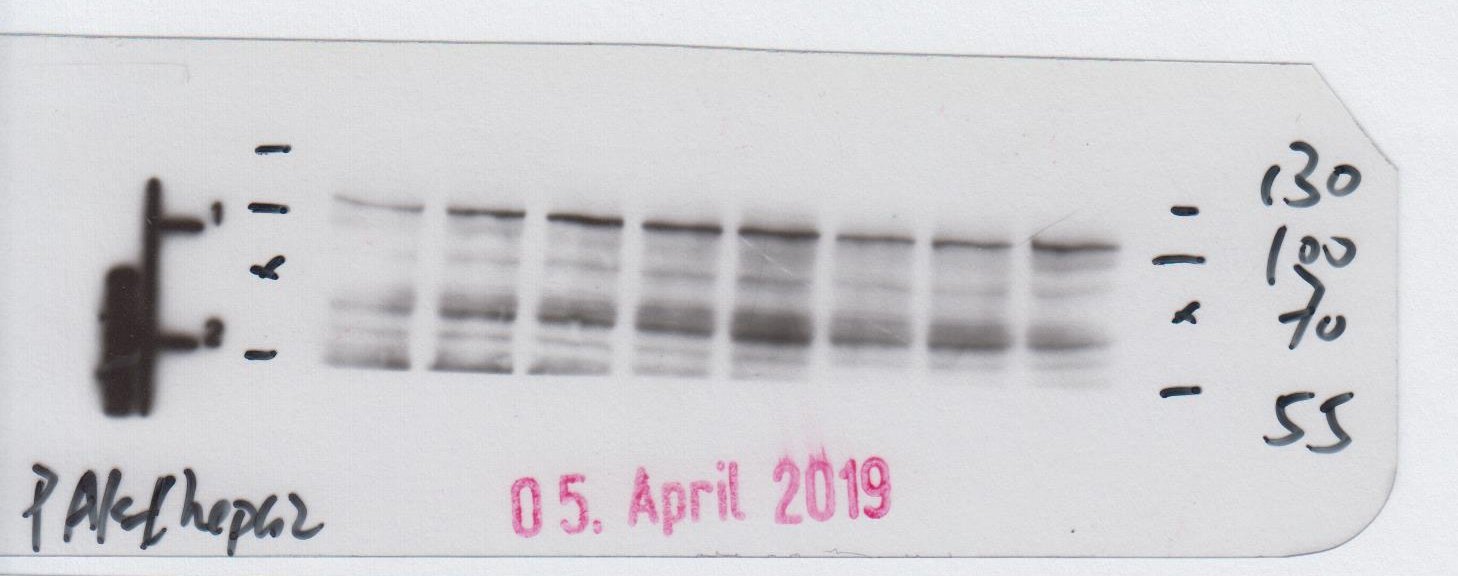

Supplement: Supplementary file 1 [file cancers-12-00615-s001.zip › cancers-670532 supplementary final/Western Blot/p-akts HEP G2.jpg]

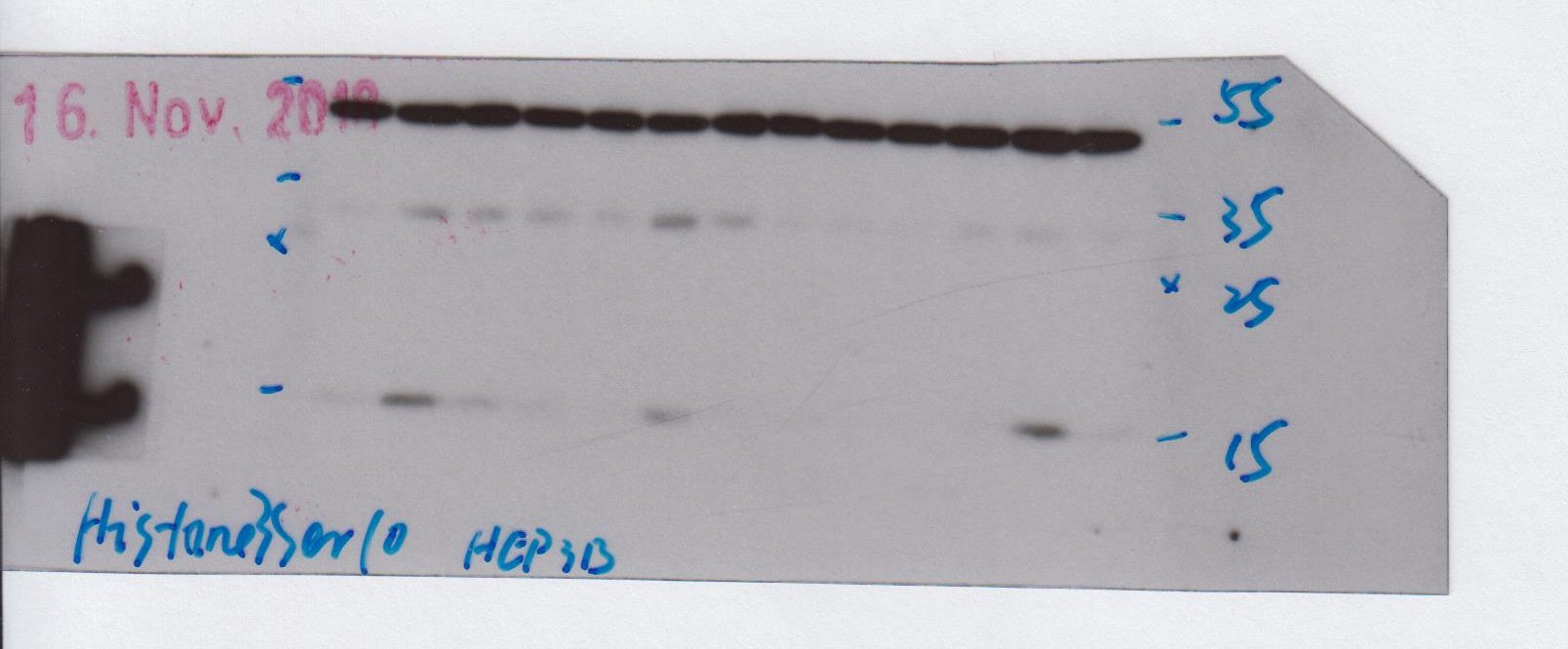

Supplement: Supplementary file 1 [file cancers-12-00615-s001.zip › cancers-670532 supplementary final/Western Blot/P-HISTONE-SER10s HEP 3B.jpg]

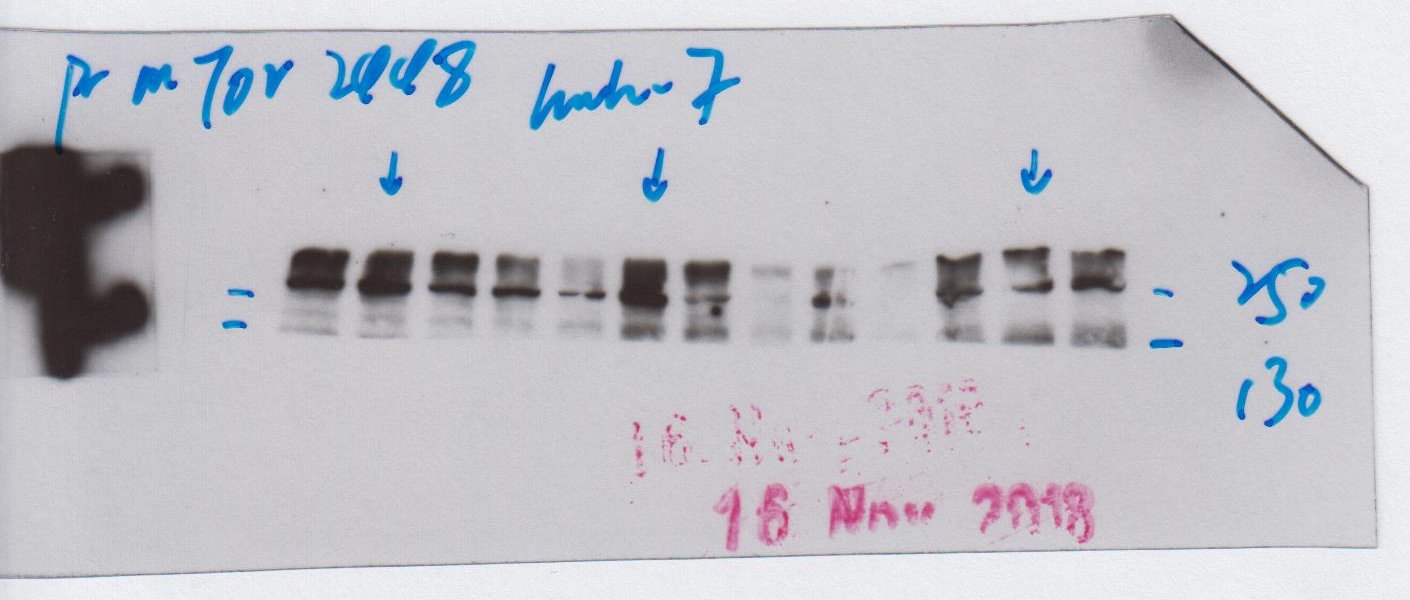

Supplement: Supplementary file 1 [file cancers-12-00615-s001.zip › cancers-670532 supplementary final/Western Blot/p-M-TORs 2448 HUH-7.jpeg.jpg]

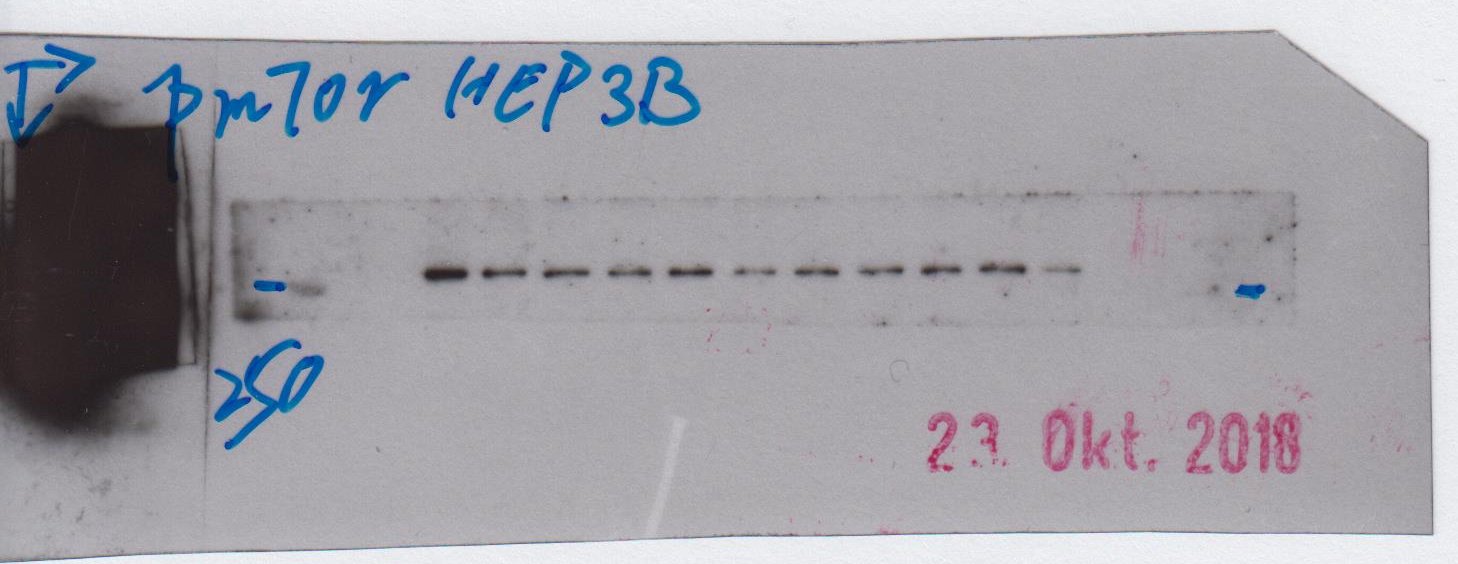

Supplement: Supplementary file 1 [file cancers-12-00615-s001.zip › cancers-670532 supplementary final/Western Blot/p-m-tors hep 3b.jpg]

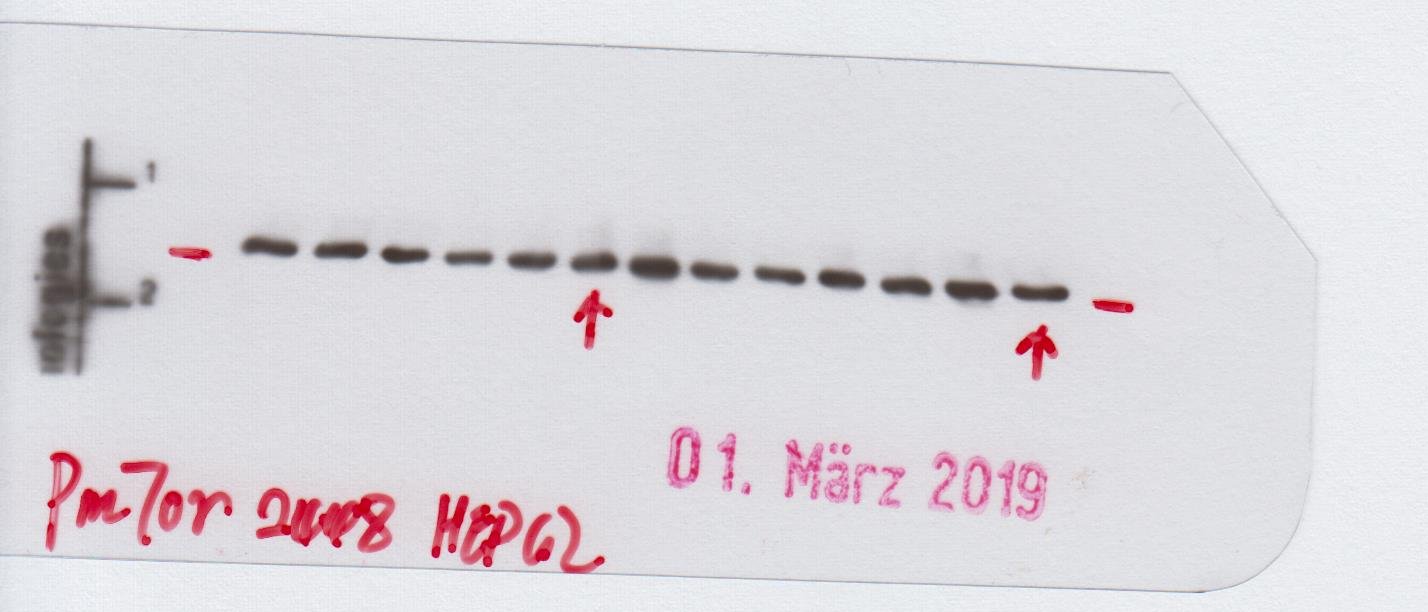

Supplement: Supplementary file 1 [file cancers-12-00615-s001.zip › cancers-670532 supplementary final/Western Blot/p-mTORs HEP G2.jpg]

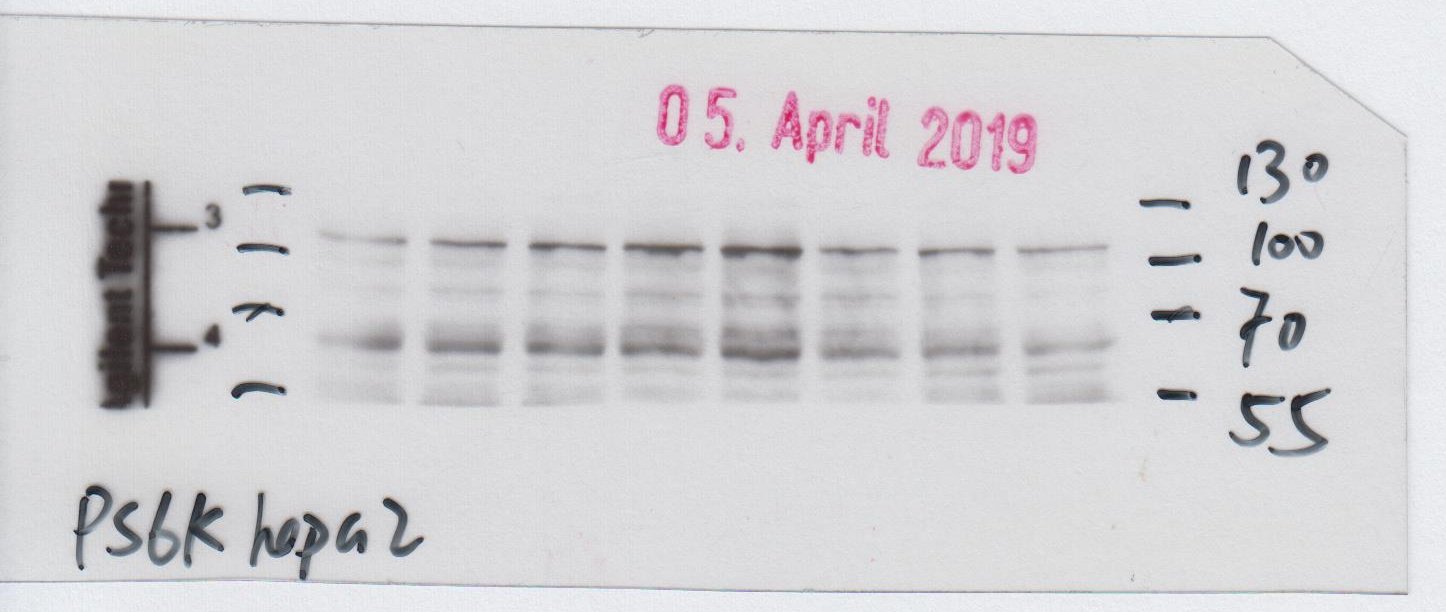

Supplement: Supplementary file 1 [file cancers-12-00615-s001.zip › cancers-670532 supplementary final/Western Blot/p-s6ks HEP G2-2.jpg]

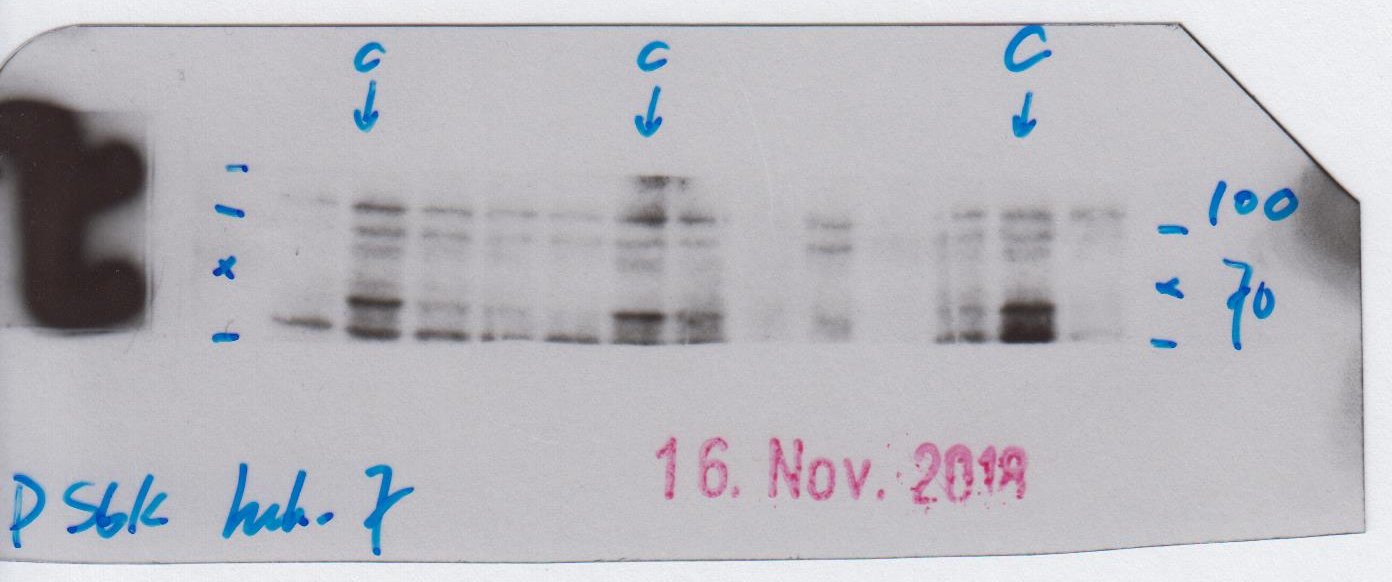

Supplement: Supplementary file 1 [file cancers-12-00615-s001.zip › cancers-670532 supplementary final/Western Blot/P-S6Ks HUH-7.jpg]

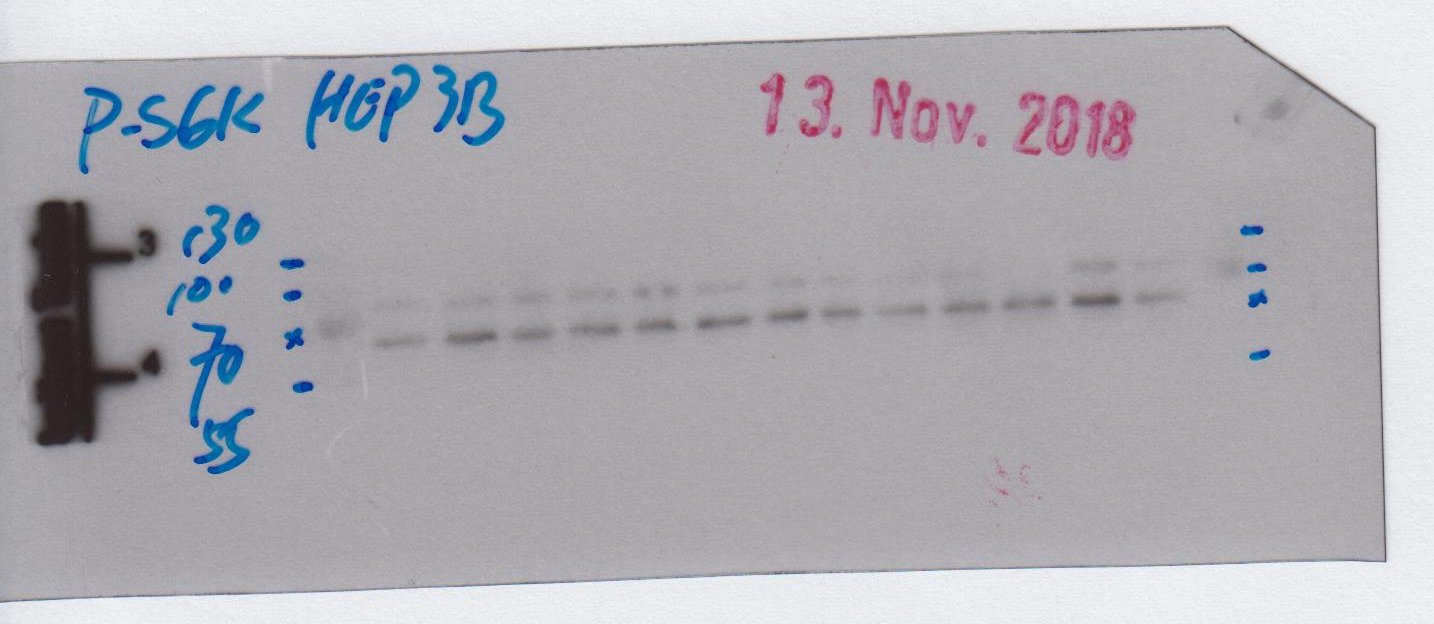

Supplement: Supplementary file 1 [file cancers-12-00615-s001.zip › cancers-670532 supplementary final/Western Blot/P-S6Ks. HEP 3Bjpg.jpg]

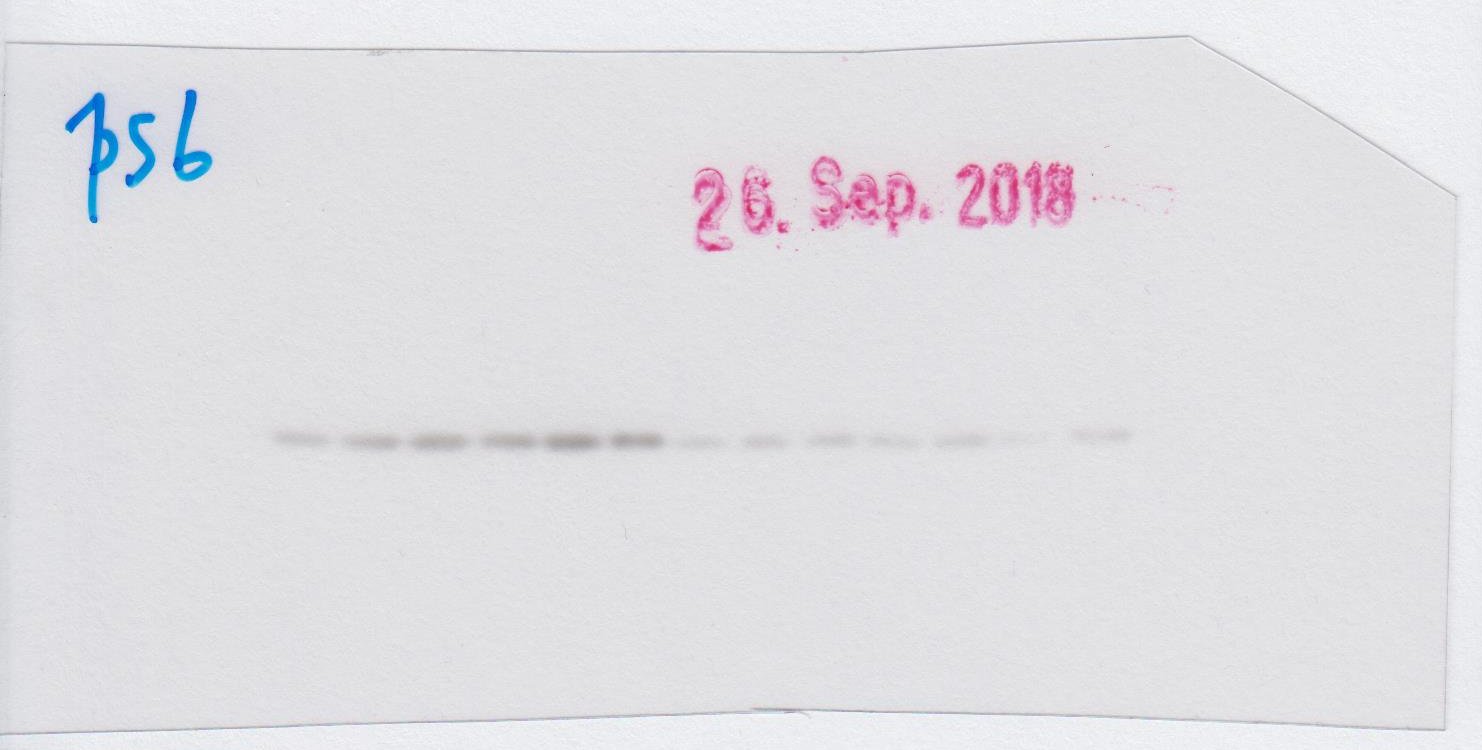

Supplement: Supplementary file 1 [file cancers-12-00615-s001.zip › cancers-670532 supplementary final/Western Blot/P-S6s HEP 3B.jpg]

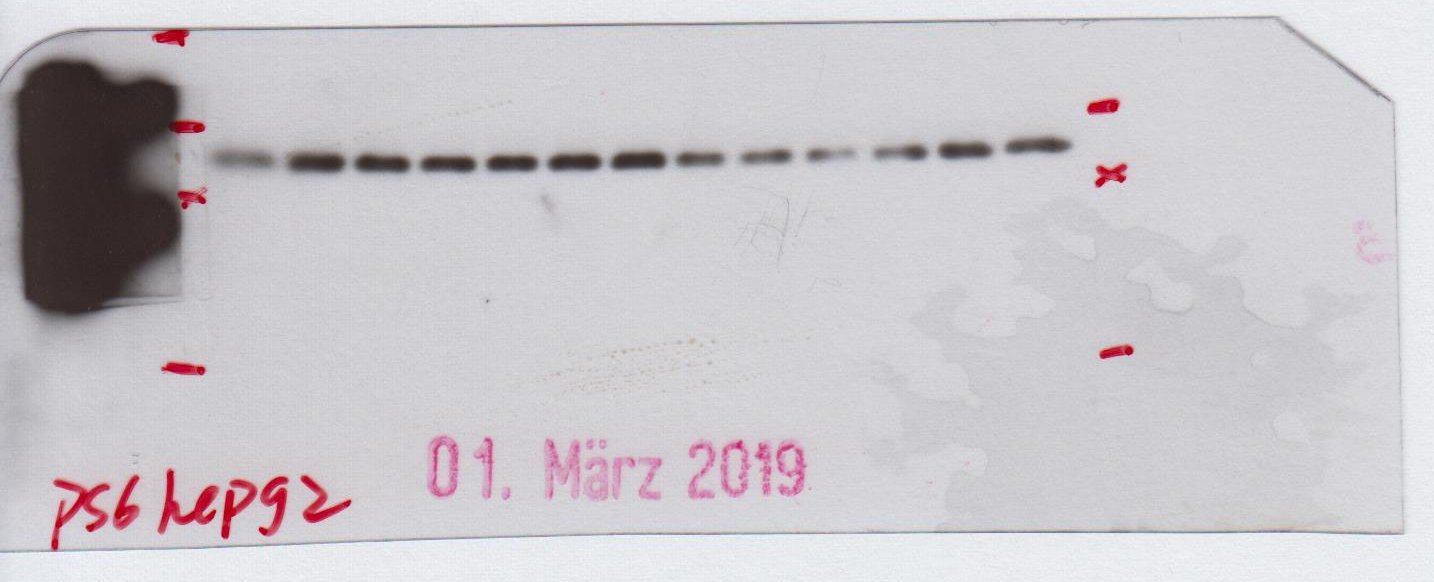

Supplement: Supplementary file 1 [file cancers-12-00615-s001.zip › cancers-670532 supplementary final/Western Blot/p-S6s HEP G2.jpg]

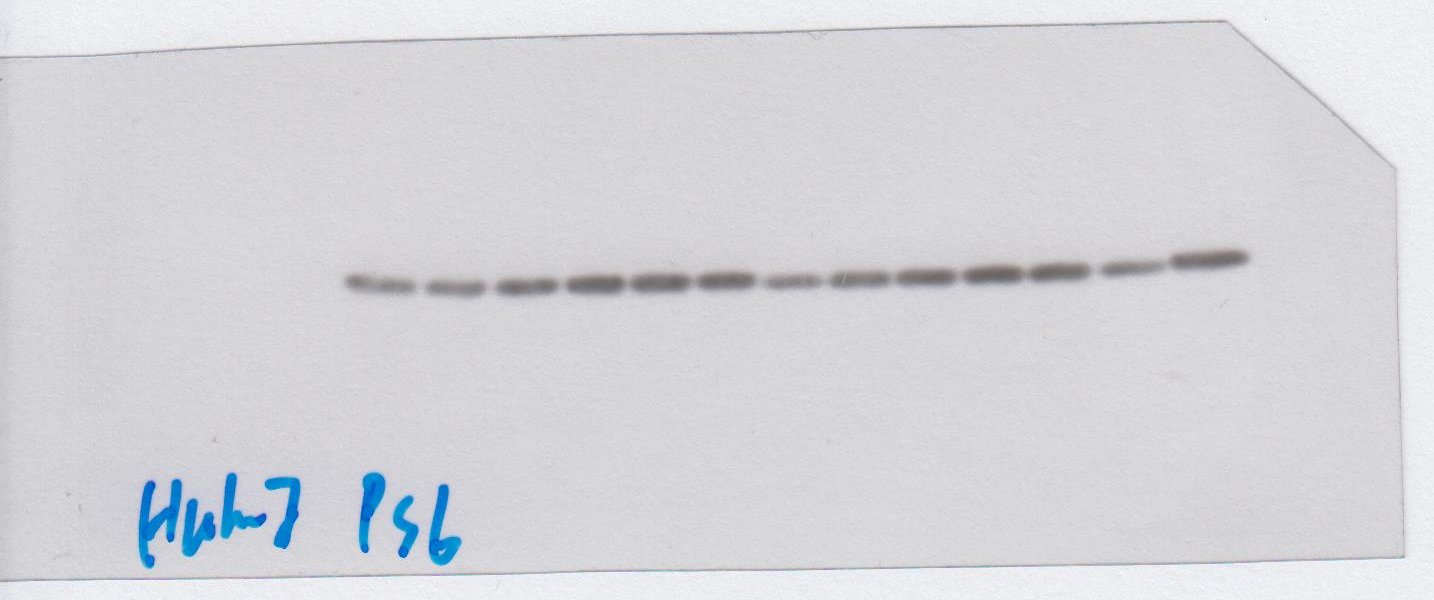

Supplement: Supplementary file 1 [file cancers-12-00615-s001.zip › cancers-670532 supplementary final/Western Blot/P-S6s HUH-7.jpg]

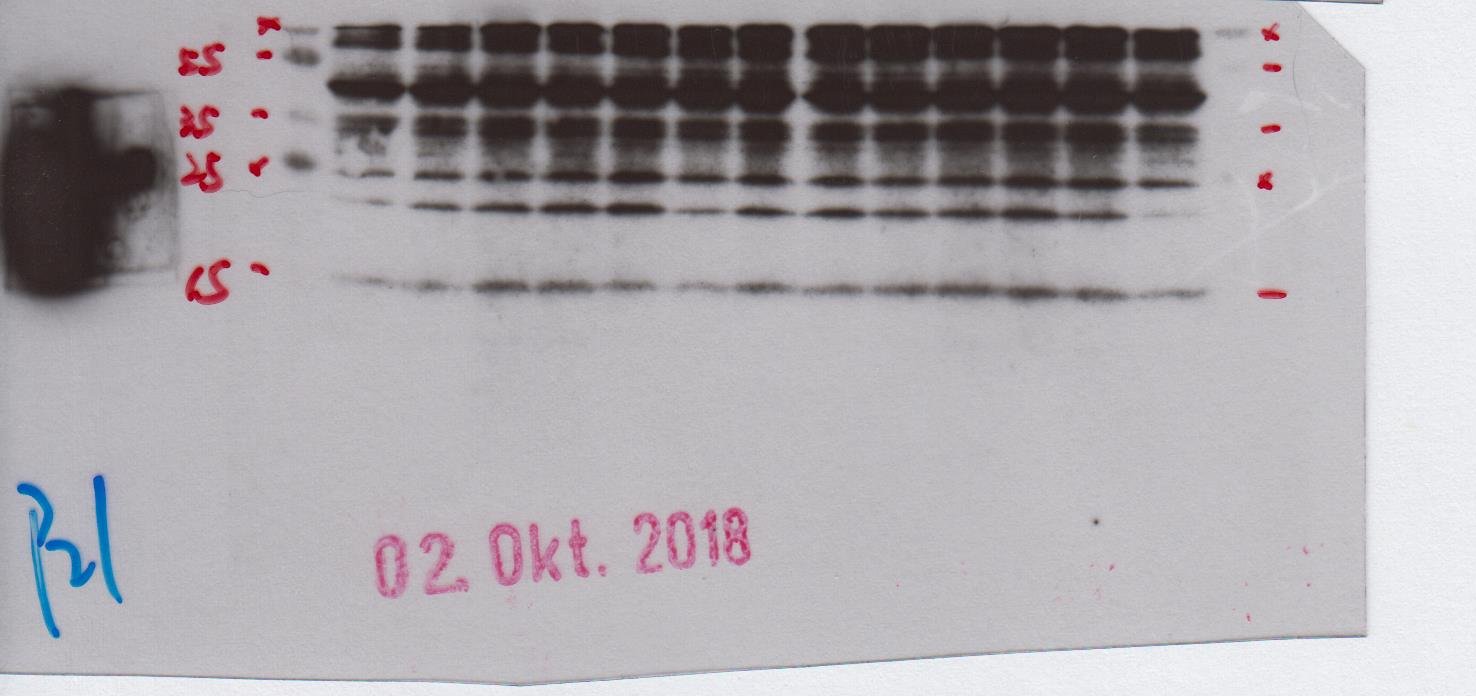

Supplement: Supplementary file 1 [file cancers-12-00615-s001.zip › cancers-670532 supplementary final/Western Blot/P21s hep 3b.jpg]

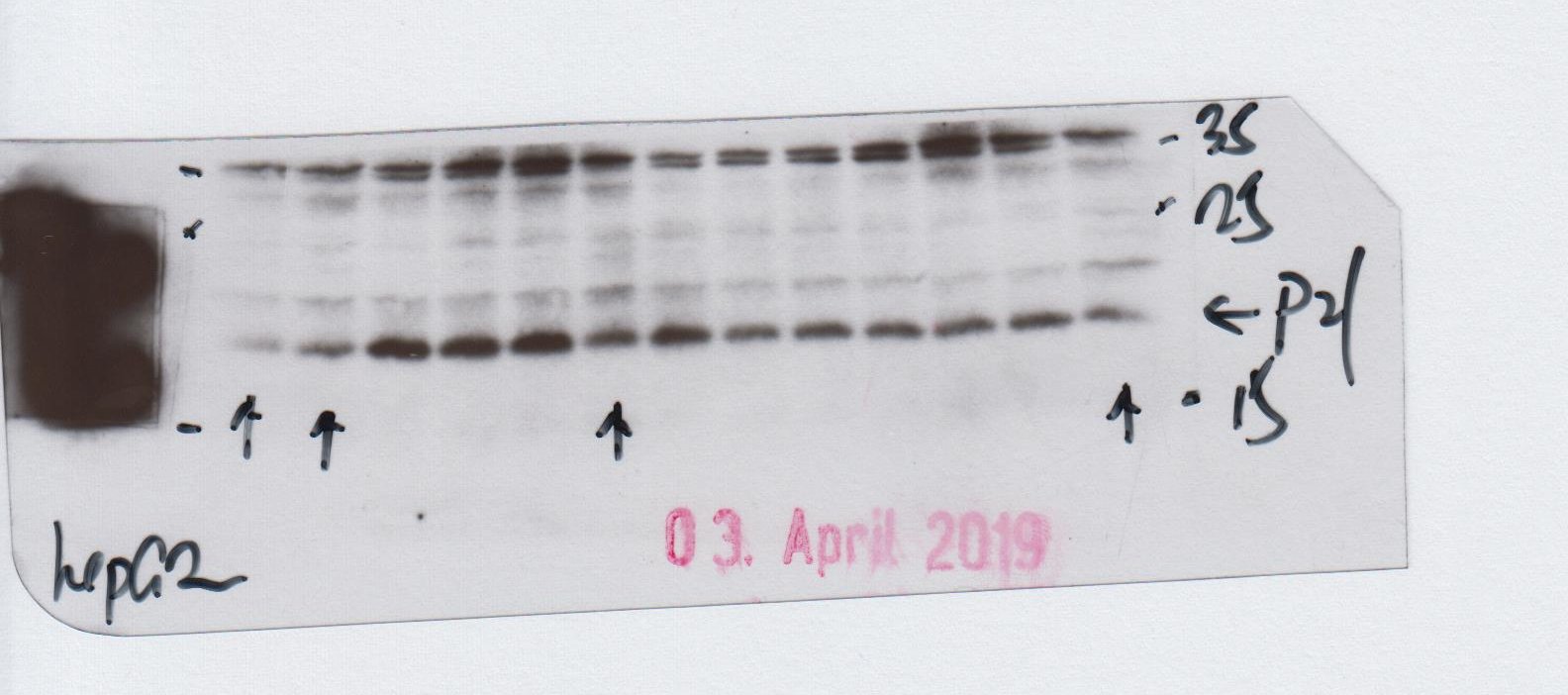

Supplement: Supplementary file 1 [file cancers-12-00615-s001.zip › cancers-670532 supplementary final/Western Blot/p21s hep g2.jpg]

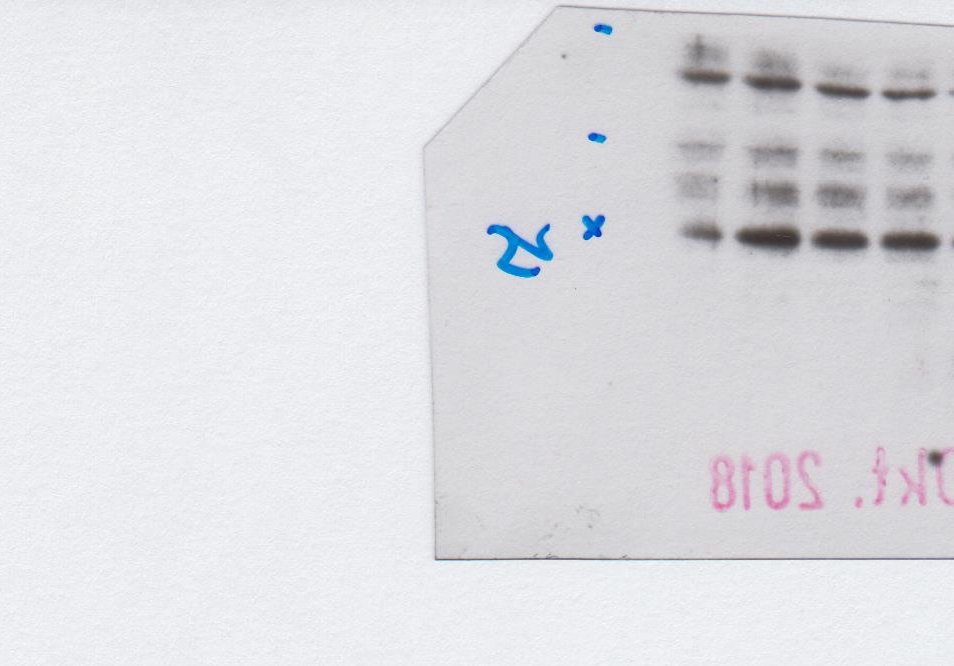

Supplement: Supplementary file 1 [file cancers-12-00615-s001.zip › cancers-670532 supplementary final/Western Blot/P21s HUH-7.jpg]

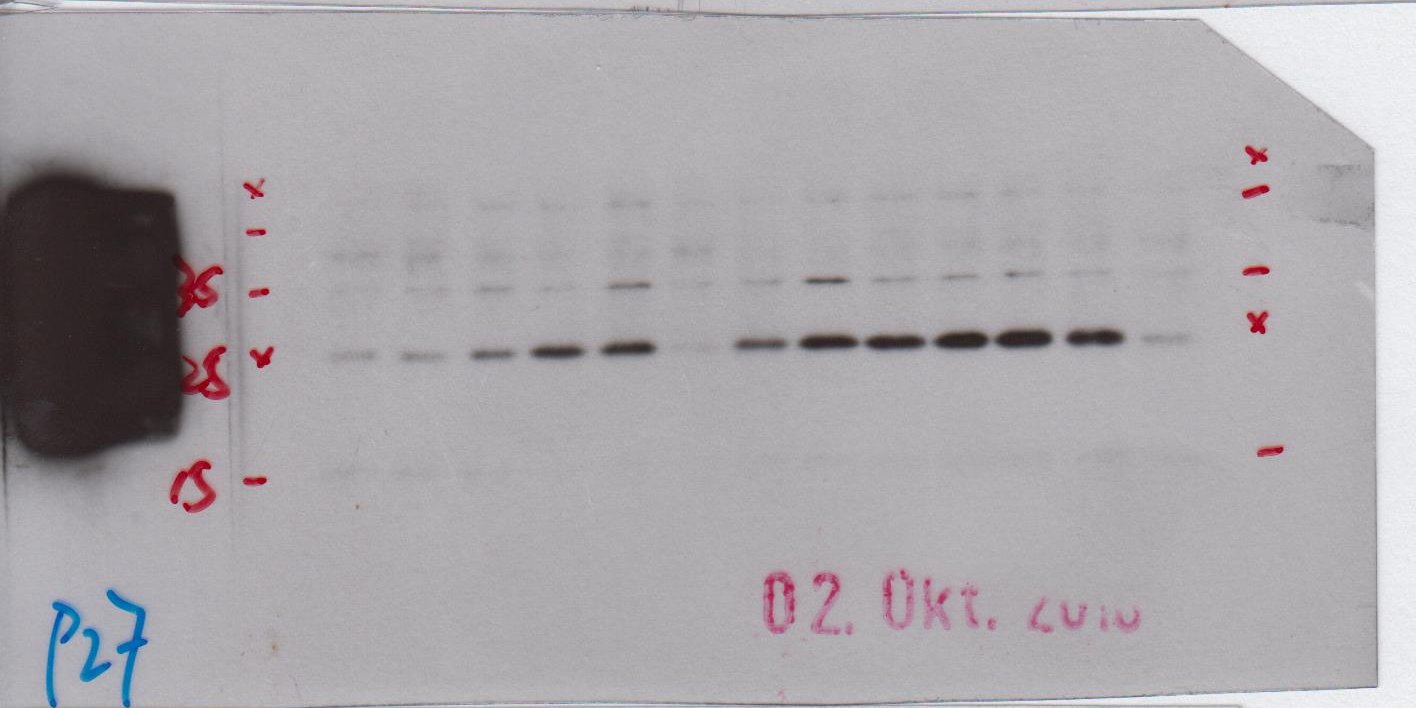

Supplement: Supplementary file 1 [file cancers-12-00615-s001.zip › cancers-670532 supplementary final/Western Blot/P27s hep 3b.jpg]

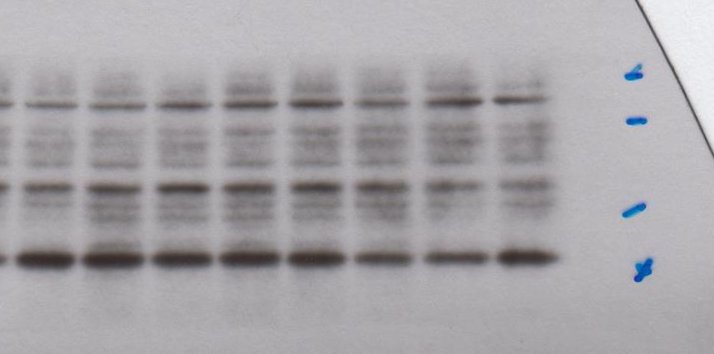

Supplement: Supplementary file 1 [file cancers-12-00615-s001.zip › cancers-670532 supplementary final/Western Blot/P27s HUH-7.jpg]

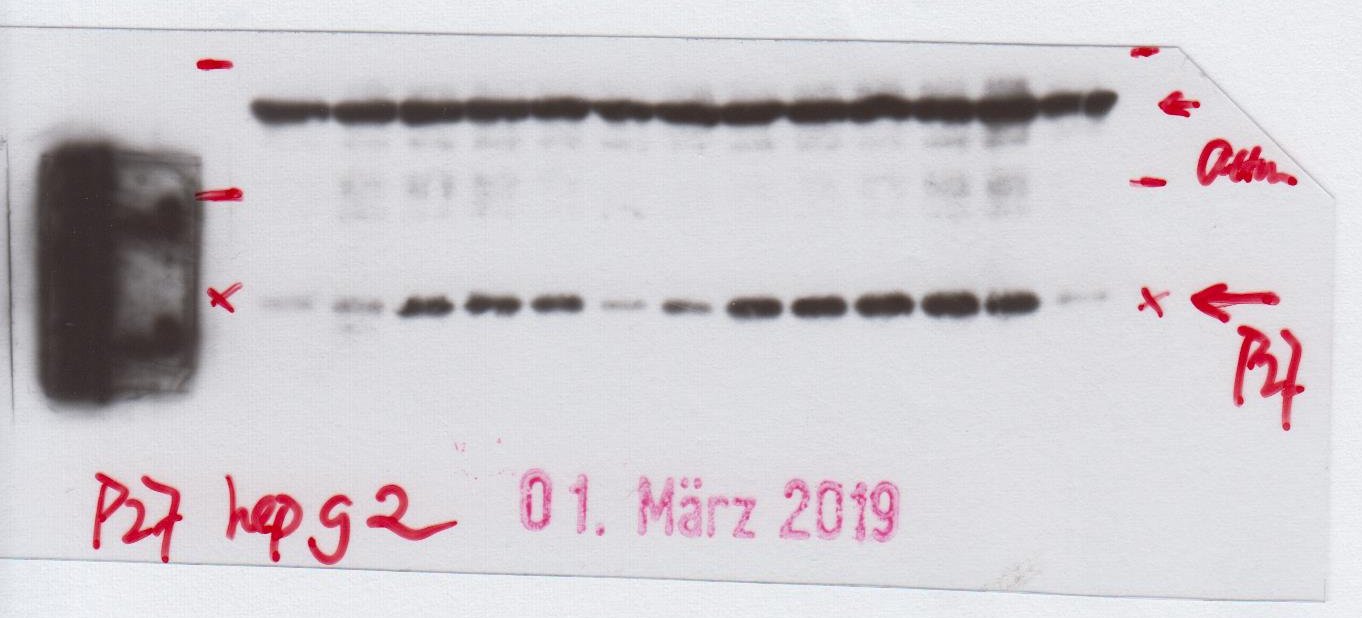

Supplement: Supplementary file 1 [file cancers-12-00615-s001.zip › cancers-670532 supplementary final/Western Blot/p27s-5 hep g2.jpg]

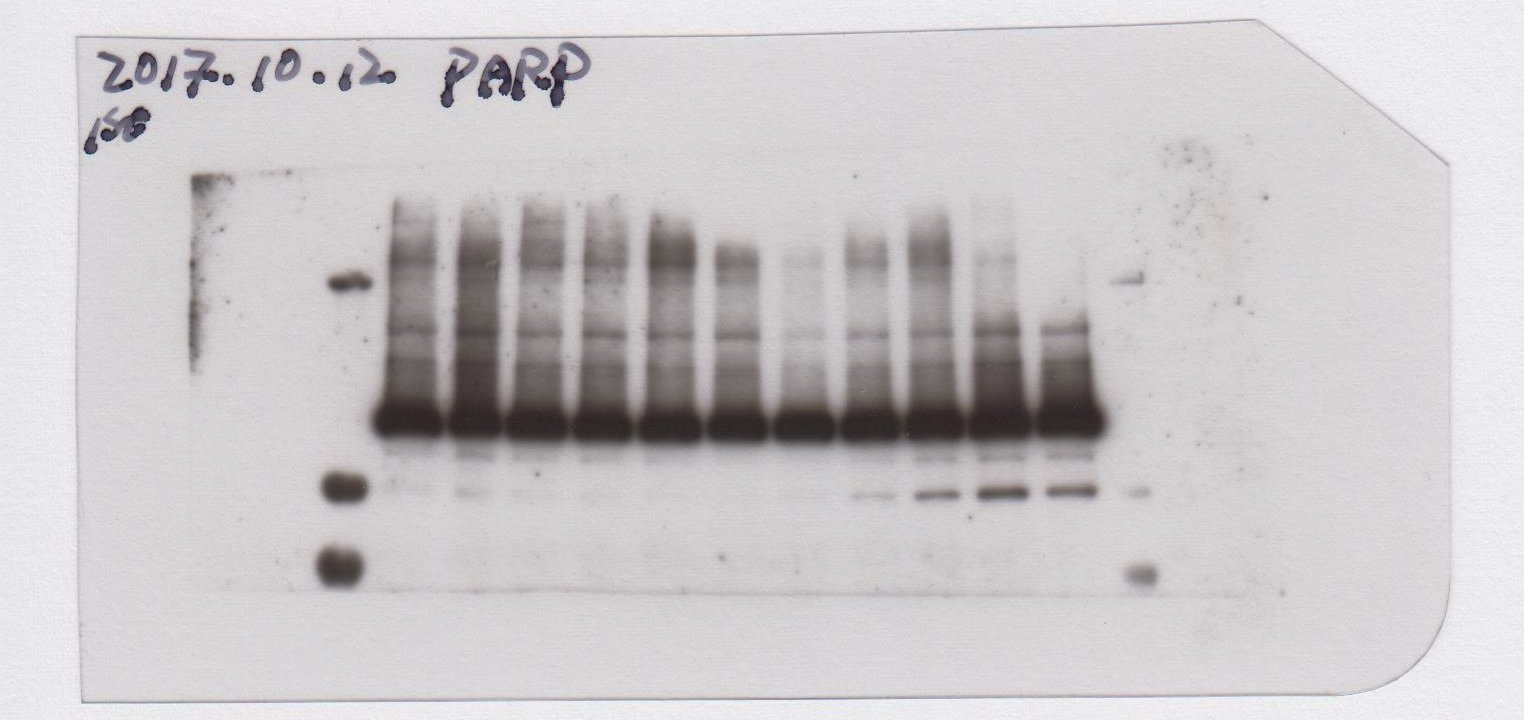

Supplement: Supplementary file 1 [file cancers-12-00615-s001.zip › cancers-670532 supplementary final/Western Blot/parps hep 3b kinetic.jpg]

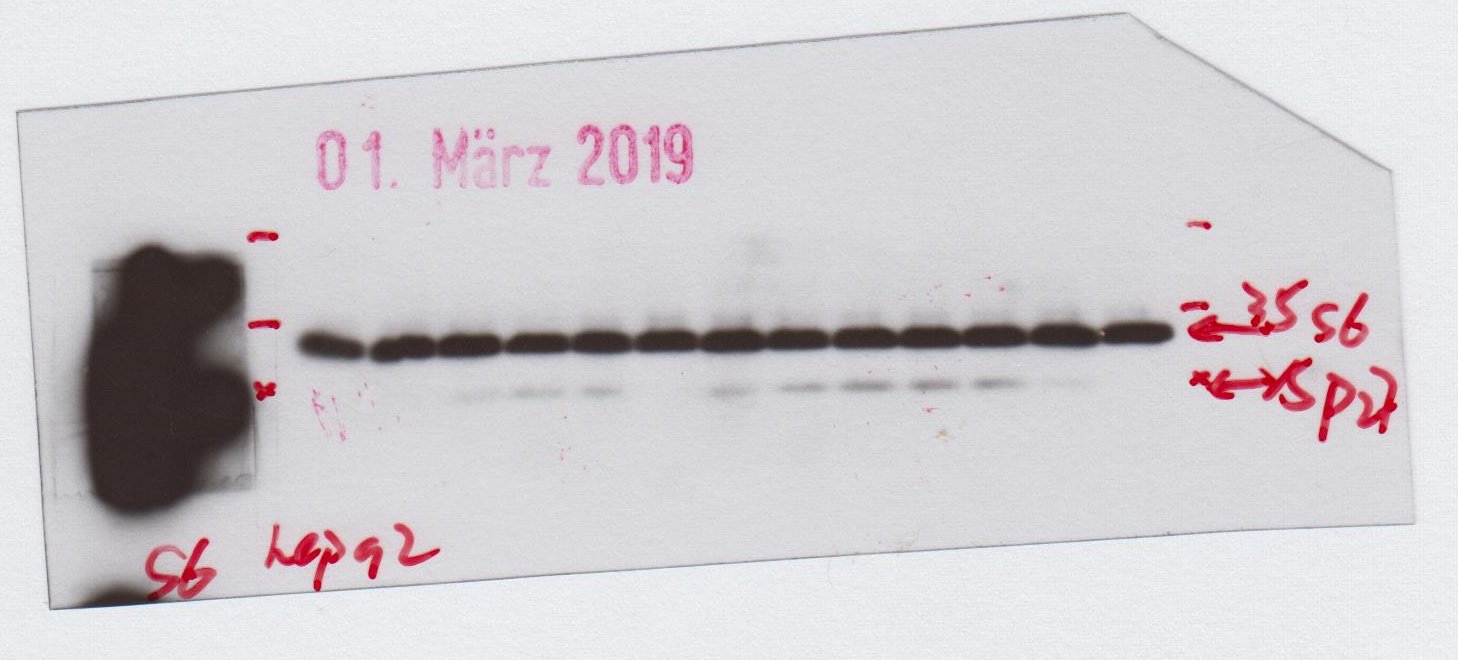

Supplement: Supplementary file 1 [file cancers-12-00615-s001.zip › cancers-670532 supplementary final/Western Blot/S6s HEP G2.jpg]

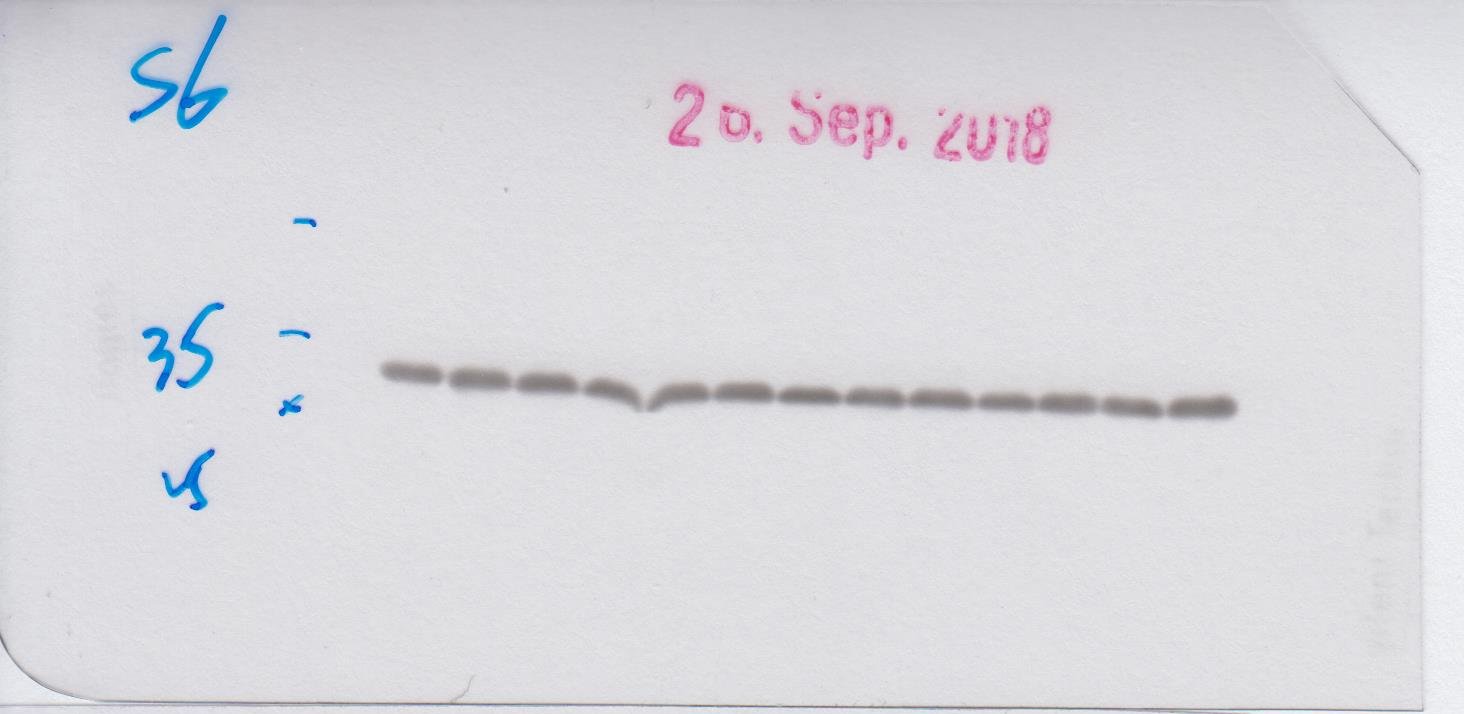

Supplement: Supplementary file 1 [file cancers-12-00615-s001.zip › cancers-670532 supplementary final/Western Blot/S6s HEP 3B.jpg]

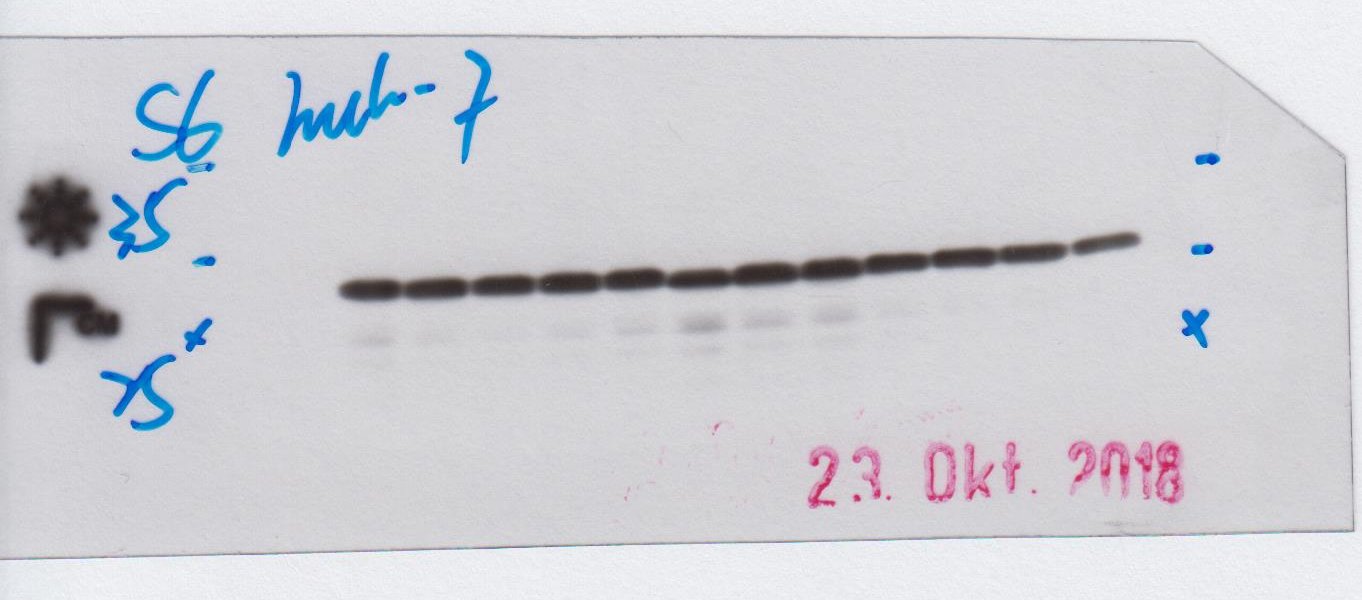

Supplement: Supplementary file 1 [file cancers-12-00615-s001.zip › cancers-670532 supplementary final/Western Blot/s6s huh-7.jpg]
